# giRAff: an automated atlas segmentation tool adapted to single histological slices

**DOI:** 10.3389/fnins.2023.1230814

**Published:** 2024-01-11

**Authors:** Sébastien Piluso, Nicolas Souedet, Caroline Jan, Anne-Sophie Hérard, Cédric Clouchoux, Thierry Delzescaux

**Affiliations:** ^1^Université Paris-Saclay, CEA, CNRS, MIRCen, Laboratoire des Maladies Neurodégénératives, Fontenay-aux-Roses, France; ^2^WITSEE, Paris, France

**Keywords:** atlas segmentation, image registration, histology, brain, mouse

## Abstract

Conventional histology of the brain remains the gold standard in the analysis of animal models. In most biological studies, standard protocols usually involve producing a limited number of histological slices to be analyzed. These slices are often selected into a specific anatomical region of interest or around a specific pathological lesion. Due to the lack of automated solutions to analyze such single slices, neurobiologists perform the segmentation of anatomical regions manually most of the time. Because the task is long, tedious, and operator-dependent, we propose an automated atlas segmentation method called giRAff, which combines rigid and affine registrations and is suitable for conventional histological protocols involving any number of single slices from a given mouse brain. In particular, the method has been tested on several routine experimental protocols involving different anatomical regions of different sizes and for several brains. For a given set of single slices, the method can automatically identify the corresponding slices in the mouse Allen atlas template with good accuracy and segmentations comparable to those of an expert. This versatile and generic method allows the segmentation of any single slice without additional anatomical context in about 1 min. Basically, our proposed giRAff method is an easy-to-use, rapid, and automated atlas segmentation tool compliant with a wide variety of standard histological protocols.

## 1 Introduction

In the last few decades, conventional histology has benefited from the expansion of light microscopy (Wilt et al., 2009; Ghaznavi et al., 2013; Milligan et al., 2019), in conjunction with the development of a wide range of biological staining techniques (Kuan et al., 2015; Kim et al., 2017; Erö et al., 2018; Tward et al., 2020; Wang et al., 2020). Cutting and acquisition protocols have become more and more sophisticated over time, providing a broad variety of procedures. This made it possible to observe the brain in an unprecedented way (Vandenberghe et al., 2016; Erö et al., 2018; Milligan et al., 2019; Tward et al., 2020). However, the resulting data remain massive and difficult to analyze for most of the labs. This is the case for the mouse brain in preclinical studies (Milligan et al., 2019).

Automated tools for analyzing these tissues, allowing the detection of biological objects and identification of the anatomical regions of interest (ROIs) to which they belong, are essential. Object segmentation has seen a tremendous upturn with the expansion of deep neural networks (Ronneberger et al., 2015; Falk et al., 2019). However, accurately identifying ROIs is still challenging and usually requires a brain atlas or expert knowledge of neuroanatomy.

As a result, many histological protocols are focused on specific anatomical regions, lesion areas, or pathological biomarkers, only on several well-chosen slices of interest within the brain (Lebenberg et al., 2010; Mesejo et al., 2012; Kim et al., 2015, 2017; Niedworok et al., 2016; Pagani et al., 2016; Renier et al., 2016; Ye et al., 2016; Dudeffant et al., 2017; Stolp et al., 2018; Zeng, 2018; Chen et al., 2019; Eastwood et al., 2019; Pallast et al., 2019; Bayraktar et al., 2020; Hérard et al., 2020; Sen et al., 2020; Song et al., 2020; Lam et al., 2022; Yee et al., 2022). It is prone to many drawbacks: this tedious work often yields non-reproducible operator-dependant results, suffers from inter- and intra-individual variability, and requires special attention in the statistical analysis design.

Digital mouse brain atlases aimed both to establish a rigorous, precise, and common reference of delineation for anatomical ROIs and, more importantly, to use them as a segmentation tool (Dauguet et al., 2007; Lein et al., 2007; Lau et al., 2008; Dubois et al., 2010; Johnson et al., 2010; Papp et al., 2014; Kuan et al., 2015; Tward et al., 2020; Wang et al., 2020).

A digital atlas being tree-dimensional (3D), the experimental volume needs to be reconstructed so that their respective dimensionality matches. But it is possible to reconstruct the organ in 3D using registration techniques when all or enough serial slices are cut and digitized (Ourselin et al., 2001; Modat et al., 2014; Agarwal et al., 2016; Niedworok et al., 2016; Fürth et al., 2018; Eastwood et al., 2019). This is the main issue to tackle, which cannot be achieved in most of the studies since protocols are not designed to yield 3D histology. One solution to overcome the lack of histological material is to use blockface photography (Toga et al., 1994) as a whole-brain template to achieve 3D reconstruction of several histological modalities of the same sample (Dauguet et al., 2007; Dubois et al., 2010; Vandenberghe et al., 2016). Indeed, 3D histology protocols are time-consuming, expensive, and neurobiologists often acquire only a limited number of slices. Therefore, the delineation of anatomical regions is mostly performed manually on the experimental data and/or the identification of their corresponding atlas slice is based on prior anatomical knowledge (Lebenberg et al., 2010; Ye et al., 2016; Iglesias et al., 2018; Pichat et al., 2018; Balakrishnan et al., 2019; Chen et al., 2019; Chon et al., 2019; Henderson et al., 2019; Pallast et al., 2019; Wu et al., 2019; Yates et al., 2019; Bayraktar et al., 2020; Hérard et al., 2020; Lam et al., 2022; Rodarie et al., 2022).

**Figure 1:**
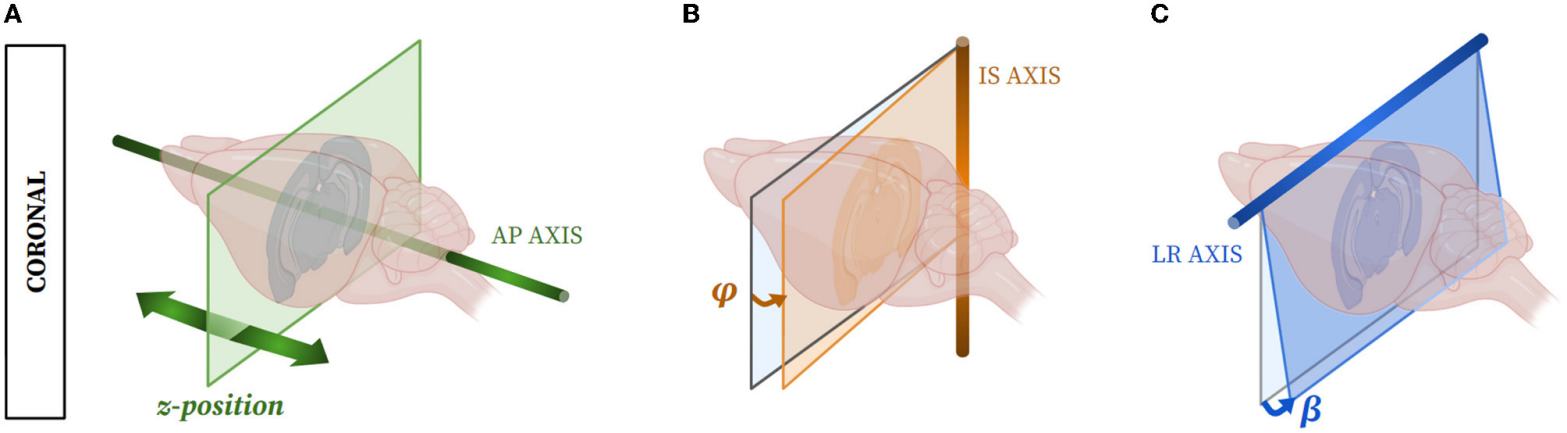
The three parameters used to exactly evaluate the 2D location of a coronal mouse brain slice within a template from an atlas: **(A)** the *z-position* along the AP axis, **(B)** the possible tilting angle φ around the IS axis, and **(C)** the possible tilting angle β around the LR axis.

Furthermore, with the expansion of artificial intelligence techniques used to automatically segment brain slices, the need for reliable annotated database creation has dramatically increased in the last 5 years (de Vos et al., 2017, 2019; Krebs et al., 2017; Li and Fan, 2017; Rohé et al., 2017; Sokooti et al., 2017; Yang et al., 2017; Balakrishnan et al., 2018, 2019; Krepl et al., 2021; Sadeghi et al., 2022; Carey et al., 2023). Hence, automated, rapid, and adaptable atlas segmentation tools are still lacking but mandatory, for instance, when dealing with the segmentation of so-called *single* brain slices (devoid of 3D reference) needing to locate the 2D plane of each slice within a 3D atlas template volume. As the mouse brain has an elongated shape, most of the studies observe mouse brains in the coronal incidence (Bohland et al., 2010; Berlanga et al., 2011; Renier et al., 2016; Vandenberghe et al., 2016; Stæger et al., 2020), and we therefore focused on this incidence. Three parameters enable the exact location of a single slice plane within the atlas volume: (1) the *z-position* of the slice along the rostro-caudal (antero-posterior, AP) axis orthogonal to the coronal plane; (2) the tilting angle φ around the dorso-ventral (infero-superior, IS) axis; and (3) the tilting angle β around the transversal (left-right, LR) axis (Figure 1). Some tools, such as cutting matrices, can be used to obtain a quasi-perfect coronal cutting incidence, i.e., with φ and β tilting angles close to zero and therefore negligible, but usually, φ and β tilting angles lead to discrepancies when comparing “real life” slices and atlas ones.

Some studies focused on identifying possible tilting angles φ and β to refine 2D-plan location within the 3D atlas space (Xiong et al., 2018), while others proposed automated methods or user-friendly softwares to handle 2D slices within a 3D space toward *z-position*-oriented estimation (Puchades et al., 2019; Tappan et al., 2019). These strategies present a more or less accurate estimation of both tilting angles and are not fully automated since they all include manual processing to estimate the *z-position*. Basically, manual processing limits the use of such methods on a large scale for the study of mouse cohorts, in particular. More recently, a feature-based method called AMaSiNe was proposed to automatically estimate *z-position*, φ, and β (Song et al., 2020). Authors evaluate them with precision (<100 μm), and segmentation results have been validated on two specific small regions only (primary visual area and dorsal lateral geniculate complex). However, the method is non-reproducible for the analysis of a single slice. In addition, the method is only robust from a minimum of three slices. Finally, a completely different approach has been proposed, using deep neural networks (Sadeghi et al., 2022; Carey et al., 2023). These methods require a large number of slices to train the network and rely on manual ground truth definition. Such estimates are prone to inter- and intra-individual error; their result is subjective and usually performed only on a relatively small part of the dataset. Moreover, the large variety of histological staining, along with the different imaging modalities, makes it very difficult to build up an exhaustive database to train a fairly generic neural network. Most of the existing methods are either very complex and not user-friendly (codes without interface) to be implemented by neurobiologists or require knowledge in neuroanatomy to be used appropriately, both greatly reducing their scope of application.

The method we propose is intended to be generic enough to be used by anyone and benefits from a user-friendly interface. The fully automatic mode we propose gives reliable results, and the user can still adjust parameters. We focused on the estimation of the *z-position* of single coronal slices. Our automated method is reproducible and can align and segment any number of single slices within a digital 3D atlas. Moreover, we developed a dedicated multi-slices extension to meet ROI-driven histological protocols, resulting in a set of slices from the same brain. Our method is based on a linear registration algorithm as well as an independent and multimodal similarity criterion. The Block Matching (BM) algorithm (Ourselin et al., 2001) was chosen as a robust strategy to register data from different modalities. This method was later included in the NiftyReg library (Modat et al., 2014) and is still well used in many applications (Niedworok et al., 2016; Iglesias et al., 2018; Balakrishnan et al., 2019; Borovec et al., 2020; Mancini et al., 2020). Normalized Mutual Information (NMI) (Studholme et al., 1998) was chosen as a robust similarity metric adapted to multimodality. This metric has proven its efficiency in many biomedical image processing applications (Jefferis et al., 2007; Geha et al., 2008; Dorocic et al., 2014; Costa et al., 2016). The idea of the method is to explore registrations between the experimental single slice and the ones from the atlas template, with increasing degrees of freedom. The NMI criterion is used to propose a generic evaluation framework of the relative similarity between slices after each step of registration. Basically, the method combines similarity information coming from *Rig*id and *Aff* ine registration, which explains the acronym we defined for this method: giRAff. We chose to refer to the Allen mouse Brain Atlas (ABA), a digital atlas widely used in neurobiology (Lein et al., 2007; Lau et al., 2008; Kuan et al., 2015). Also, we focused on histological slices covering the cortex, excluding the main olfactory bulb and the cerebellum. Most of the biological samples come from healthy subjects, but we also present some preliminary results on a pathological subject (Alzheimer's disease mouse model).

In addition, high-performance computing strategies were used to reach our goal of segmenting a large number of histological slices. Indeed, as registrations have a relatively high computational cost, calculations were distributed on hundreds of CPU cores through the dedicated tool SomaWorkflow (Laguitton et al., 2011). Finally, to make the method easy-to-use, it was implemented within the user-friendly open-source software interface BrainVISA (Cointepas et al., 2001; Lebenberg et al., 2010).

## 2 Materials and methods

### 2.1 Materials

#### 2.1.1 Digital mouse brain atlas

In this study, we used the template and atlas from the Allen mouse Brain Atlas (ABA) (© 2015 Allen Institute for Brain Science. Allen Brain Atlas API. Available from: brain-map.org/api/index.html). It is composed of two perfectly aligned datasets: a template that represents the average anatomy of the mouse brain and labels that represent the theoretical delimitation of anatomical regions delineated by an expert on the template data. This template was built as an average autofluorescence of 1,675 serial two-photon tomography C57Bl/6J mouse brains, for which we considered each coronal slice *T*_*a*_ ϵ *B* independently. *B* is the ensemble of slices describing the template volume considered a succession of independent slices in a given incidence (here coronal). The slice thickness is *e*_*t*_ = 100 μm and the in-plane resolution is 10 × 10 μm^2^. In this study, we aimed to register 2D template images onto experimental histological slices. The purpose is to identify in the single slice of interest all the regions defined in the ABA reference corresponding slice.

#### 2.1.2 Histological dataset

In this study, we aim to segment single 2D mouse brain coronal slices *I*_*r*_, digitized from two different and independent histological modalities (see Supplementary material S0 for detailed protocols).

The first modality (so-called *autofluorescence*) is the autofluorescence of six clarified half mouse brains (M_1_-M_6_) imaged using light sheet fluorescence microscopy (Renier et al., 2016) that are considered as a succession of 2D coronal slices *I*_*r*_ devoid of cutting artifacts by nature. Those data were initially acquired with a resolution of 4 × 4 × 3 μm^3^ and resampled to 25 × 25 × 100 μm^3^ to generate a standard histological dataset.

The second modality (so-called *cresyl violet*) is cresyl violet-based Nissl staining of seven mouse whole brains produced in our laboratory (Vandenberghe et al., 2016) cut in the coronal incidence (*I*_*r*_) using a microtome and digitized with a flatbed scanner. This second dataset includes six C57Bl/6J wild-type mouse brains (M_7_-M_12_) and one APP/PS1dE9 amyloid mouse brain (M_13_), a transgenic mouse model of Alzheimer's disease (Dudeffant et al., 2017). The slice thickness is *e*_*r*_ = 20 μm (one every four slices) and the in-plane resolution is 25 × 25 μm^2^. Regarding the cutting protocol, no specific instructions were given to prevent tilting angles. The cresyl violet data arose from our laboratory routine protocols in conventional histology.

### 2.2 Methods

#### 2.2.1 Preprocessing

The template slices were first resampled in 2D to make the pixel size identical to the experimental data. Thus, the same number of pixels were used in the registration process by BM.

All images were resampled at 25 × 25 μm^2^ for registration. This resampling was chosen as a compromise between a pixel size small enough to apply the registration in a reasonable time and large enough to preserve sufficient details in the image for the registration algorithm. In such a conventional histology study, data are commonly resampled at an in-plane resolution of 25 × 25 μm^2^ (Renier et al., 2016) or 50 × 50 μm^2^ (Song et al., 2020).

The template slices were also manually centered to correspond to the experimental images. This gave a good initialization, minimizing the amplitude of the displacements induced by the registration process and maximizing the tissue overlap at an equivalent field of view.

#### 2.2.2 The giRAff method for one single slice

The giRAff method estimates the *z-position* of a single mouse brain slice within an atlas volume at a given incidence and considers zero or negligible tilting angles. This estimated *z-position* is associated with a transformation resulting from the registration between the corresponding template slice at the *z-position* and the experimental slice. The estimation of the *z-position* is given by the optimum of a similarity criterion estimated between the experimental slice considered and a set of registered slices from the template. The final result is the atlas segmentation of the single experimental slice considered through the registered and identified corresponding label slice.

The method is based on the atlas from the ABA and the linear registration method by Block Matching (BM) based on the Crossed Correlation (CC) similarity metric with the default parameters given by Ourselin et al. (2001), designed for such a histological dataset. Normalized Mutual Information (NMI) is the independent metric that quantifies the similarity between the registered template slices and the experimental single slice considered in pairs.

Given an incidence (here coronal), consider *I*_*r*_ an experimental single slice to be segmented by atlas and *T*_*a*_ a slice from an ensemble *B* of slices describing the template volume considered as a succession of independent slices, such as {*T*_*a*_ ϵ *B*}. Let *L*_*a*_ be a slice from an ensemble *A* of slices describing the labels considered as a succession of independent slices, such as {*L*_*a*_ ϵ *A*}, *A* and *B* being in the same geometry and perfectly aligned. Let *N* be the number of considered template slices in a given incidence (along the AP, IS, or LR axis), *a* ϵ ℕ^*^, going from 1 to *N*, the considered template slice number. Each template slice (from *B*) has its corresponding slice containing the labels (from *A*). Assume *z* = â, the estimated position of the slice *I*_*r*_ within the template, i.e., the corresponding slice containing the labels. We chose to register template images (test) onto the experimental data (reference) to preserve the native geometry of the single slice (experimental) given as input by a user. Hence, labels will be mapped in the end onto the single slice to match its initial configuration.

**Figure 2:**
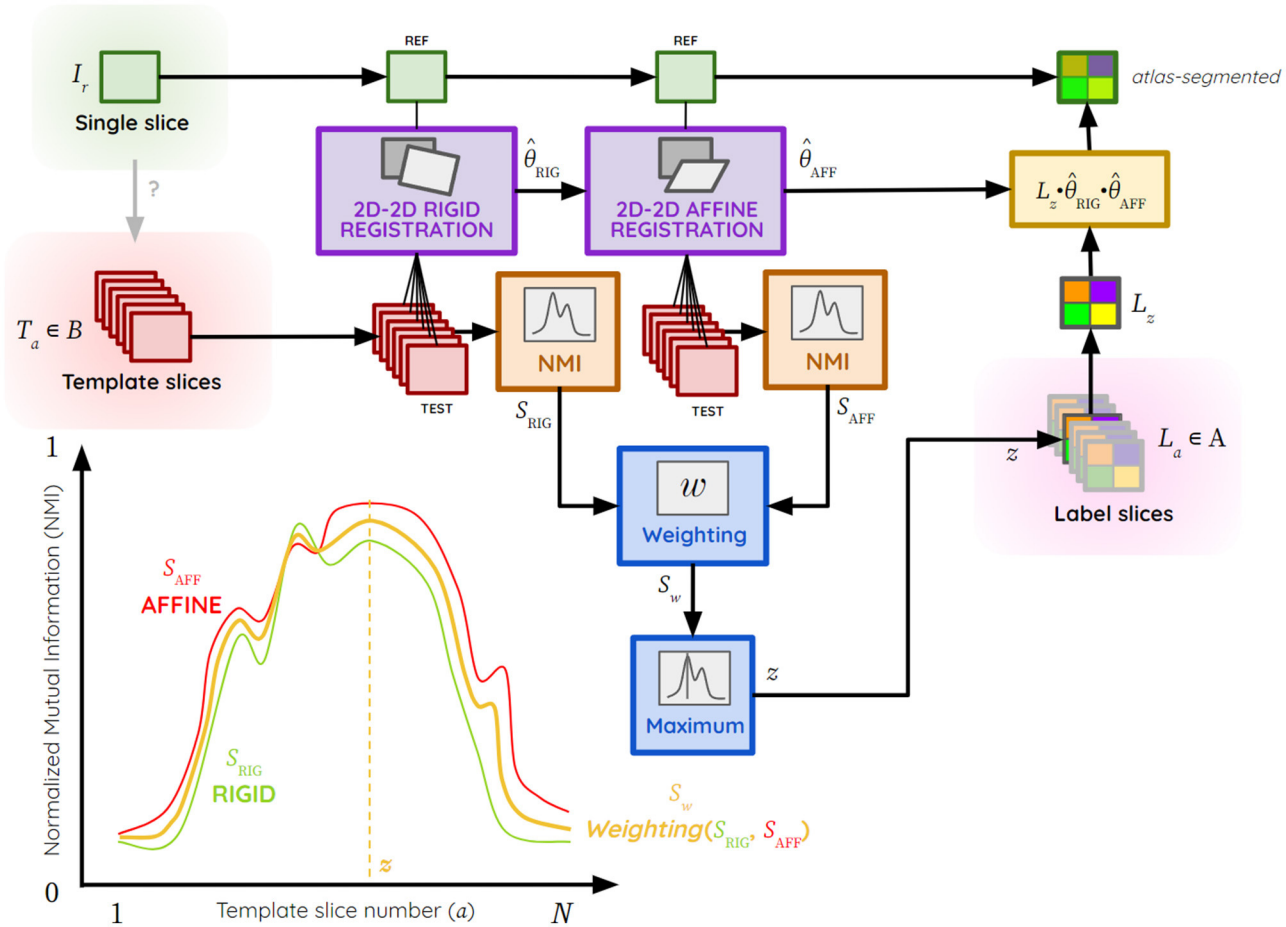
Atlas segmentation process of a single brain slice *I*_*r*_ by the giRAff method using template slices *T*_*a*_ ϵ *B*, theoretical example of NMI curve plots with *S*_RIG_ (green), *S*_AFF_ (red) and *S*_*w*_ (yellow), *z* the template slice number corresponding to a slice *I*_*r*_ estimated by the giRAff method, ${\hat{\theta }}_{\text{RIG}}$ the rigid transformation, ${\hat{\theta }}_{\text{AFF}}$ the affine transformation, *B* the ensemble of template slices, and *L*_*a*_ ϵ *A* the ensemble of label slices matching the template ones.

The exploratory process for each image *T*_*a*_ ϵ *B* is carried out in three steps (Figure 2), with RIG and AFF representing the rigid and affine transformation space, respectively:

(1) Rigid registration using BM (transformation ${\hat{\theta }}_{\text{RIG}}$) between *I*_*r*_ (reference) and *T*_*a*_ (test) from *B*, followed by an NMI similarity calculation *S*_RIG_ between the registered image $T_{a}\cdot {\hat{\theta }}_{\text{RIG}}$ and *I*_*r*_,
  (1) $$\begin{array}{l} S_{\text{RIG}}\left(I_{r},T_{a};{\hat{\theta }}_{\text{RIG}}\right)=\text{NMI}\left(I_{r},T_{a}○{\hat{\theta }}_{\text{RIG}}\right) \end{array}$$
  $$\begin{array}{l} \text{with }{\hat{\theta }}_{\text{RIG}}=\underset{\theta_{\text{RIG}}\in \text{RIG}}{argmax}\left(\text{CC}\left(I_{r},T_{a}○\theta_{\text{RIG}}\right)\right) \end{array}$$
(2) Affine registration using BM (transformation ${\hat{\theta }}_{\text{AFF}}$) between *I*_*r*_ (reference) and $T_{a}\cdot {\hat{\theta }}_{\text{RIG}}$ (test) registered in rigid (initialization), followed by NMI similarity calculation *S*_AFF_ between the registered image $T_{a}\cdot {\hat{\theta }}_{\text{RIG}}\cdot {\hat{\theta }}_{\text{AFF}}$ and *I*_*r*_,
  (2) $$\begin{array}{l} S_{\text{AFF}}\left(I_{r},T_{a};{\hat{\theta }}_{\text{AFF}}\right)=\text{NMI}\left(I_{r},T_{a}○{\hat{\theta }}_{\text{RIG}}○\;{\hat{\theta }}_{\text{AFF}}\right) \end{array}$$
  $$\begin{array}{l} with\;{\hat{\theta }}_{\text{AFF}}=\underset{\theta_{\text{AFF}}\in \text{AFF}}{argmax}\left(CC\left(I_{r},T_{a}○\theta_{\text{RIG}}○\theta_{\text{AFF}}\right)\right) \end{array}$$
(3) Calculation of the weighted average *S*_*w*_ from the two similarity values *S*_RIG_ and *S*_AFF_:
  (3) $$\begin{array}{l} S_{w}\left(I_{r},T_{a},{\hat{\theta }}_{\text{RIG}},{\hat{\theta }}_{\text{AFF}}\right)=\left(1-w\right)S_{\text{RIG}}\left(I_{r},T_{a};{\hat{\theta }}_{\text{RIG}}\right)\\ +wS_{\text{AFF}}\;\left(I_{r},T_{a};{\hat{\theta }}_{\text{RIG}},{\hat{\theta }}_{\text{AFF}}\right) \end{array}$$

with 0 ≤ *w* ≤ 1 the rigid-affine weighting.

From the weighted average *S*_*w*_ calculated for each slice *T*_*a*_ from *B*, a search of the maximum of similarity is performed to determine the slice number *z* from *B*, maximizing this similarity criterion from the *N* template slices:


(4) $$\begin{array}{l} z\left(I_{r},B\right)=\underset{T_{a}\in B}{argmax}\left(S_{w}\left(I_{r},T_{a},{\hat{\theta }}_{\text{RIG}},{\hat{\theta }}_{\text{AFF}}\right)\right) \end{array}$$

Thus, the result of the giRAff method can be summarized as follows:


(5) $$\begin{array}{l} \text{giRAff}\left(I_{r},B\right)=\left(z,{\hat{\theta }}_{\text{RIG}},{\hat{\theta }}_{\text{AFF}}\right) \end{array}$$

The rigid and affine transformations ${\hat{\theta }}_{\text{RIG}}$ and ${\hat{\theta }}_{\text{AFF}}$ estimated by BM are successively applied at the slice *L*_â_ from the atlas at the position â = *z* to superimpose the registered image containing the labels ${\hat{L}}_{â}$ on *I*_*r*_, the experimental image.


(6) $$\begin{array}{l} {\hat{L}}_{â}\left(z,{\hat{\theta }}_{\text{RIG}}\;,{\hat{\theta }}_{\text{AFF}}\right)=L_{z}○{\hat{\theta }}_{\text{RIG}}○{\hat{\theta }}_{\text{AFF}} \end{array}$$

The transformation matrices ${\hat{\theta }}_{\text{RIG}}$ and ${\hat{\theta }}_{\text{AFF}}$ are applied to the slice *L*_â_ with the nearest neighbor interpolation to preserve the initial values of the labels. The experimental single slice *I*_*r*_ is then automatically segmented by the ABA. Quantitative region-based analysis can then be carried out on it thanks to the method.

#### 2.2.3 The giRAff_m_ extension for a multi-slices case

##### 2.2.3.1 Relative scaling factor between brain samples

Two mouse brains are often considered to be roughly the same size, but this is not the case in practice. Two factors influence the size of the organ, in particular: inter-individual variability (natural) and the extraction, cutting, and staining protocol to which the sample is subjected before analysis (non-natural).

**Figure 3:**
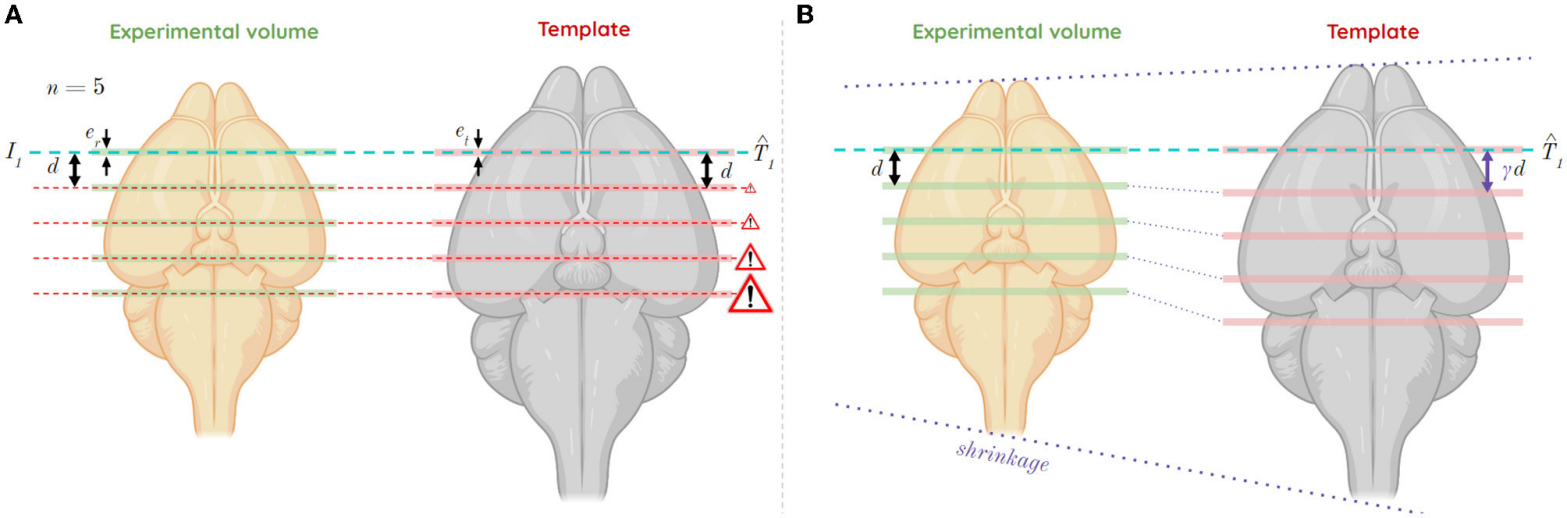
Shrinkage case of the experimental volume compared to the template volume, and its impact in a multi-slices study in the coronal incidence along the AP axis, for slice thicknesses *e*_*r*_ and *e*_*t*_ from the experimental and template volumes, respectively, as well as an inter-slice distance *d* between each considered slice from the experimental volume. **(A)** Study of five slices from a first *T*_1_ estimate without consideration of shrinkage between volumes (mismatch). **(B)** Study of five slices from a first estimate *T*_1_ considering the shrinkage between volumes using RSF γ (correction).

Let us consider a multi-slices case, i.e., a series of single histological slices from the same mouse brain not enabling its 3D reconstruction. Let *d*_*r*_ be the constant inter-slice distance between single slices from the experimental volume. Let *d*_*t*_ be the inter-slice distance between slices from the template volume. To realistically estimate the corresponding distance *d*_*r*_ depending on *d*_*t*_ in the template, the differences in brain volumetrics must be taken into account. Not taking them into account would lead to a deviation in the estimation of successive slice positions (Figure 3A). For this reason, a *relative scaling factor* γ (RSF) was introduced, which reflects the size difference between an experimental brain and the atlas template volume on the axis to which the considered incidence plane is orthogonal (Figure 3B). The affine registration automatically corrects the scaling factors in the other two directions (α, β) relative to the plane of incidence considered. This RSF γ is relative because no modality accounts for an absolute reference geometry: it is relative between two modalities.

Thus, assume:


$$\begin{array}{l} \{\begin{array}{ccc} 0<\gamma <1 & \;\Leftrightarrow & \;\text{shrinkage}\\ \gamma =1 & \;\Leftrightarrow & \;\text{same size}\\ 1<\gamma <+\infty & \;\Leftrightarrow & \;\text{enlargment} \end{array} \end{array}$$

The distances *d*_*r*_ and *d*_*t*_ are defined as a function of γ and the two slice thicknesses *e*_*r*_ for the experimental data and *e*_*t*_ for the template data:


(7) $$\begin{array}{l} d_{r}=\gamma \frac{e_{r}}{e_{t}}d_{t} \end{array}$$

Let *r* ϵ N^*^ be the slice number from the experimental data ranging from *r* = 1 to *r* = *M*, and *t* the slice number estimated to be the most similar in the template by the giRAff method, ranging from *t* = 1 to *t* = *N*. The equation of the affine line linking slice numbers from the two volumes to each other can thus be deduced:


(8) $$\begin{array}{l} \hat{t}\left(r\right)=\gamma \frac{e_{t}}{e_{r}}\left(r-1\right)+{\hat{t}}_{1} \end{array}$$

with $\hat{t_{1}}$ the y-intercept corresponding to the result of the giRAff method applied to the first slice of the experimental volume studied (*r* = 1).

##### 2.2.3.2 Operating mode

For each considered experimental single slice from a multi-slices set, similarity values with all the template slices are computed by the giRAff method and stored in a list *s*_*w*_ (see Equation 3, which is applied for each slice *T*_*a*_ ϵ B). The multi-slices analysis aims to bring each of these lists into a single referential to pool their contribution.

Assume (*u*_*s*_${)}_{s\epsilon N^{*}}$ an arithmetic series determining the first template slice number to be tested in the case of a multi-slices study and (*v*_*s*_${)}_{s\epsilon N^{*}}$ an arithmetic series determining the last template slice number to be tested in the case of a multi-slices study, we then have:


(9) and (10) $$\begin{array}{l} u_{s}=u_{1}+\frac{d_{t}}{e_{t}}\left(s-1\right)\;and\;v_{s}\;=\;N-\frac{d_{t}}{e_{t}}\left(n-s\right)\;\left(9\right)\;and\;\left(10\right) \end{array}$$

with *u*_1_ = 1 corresponding to the first template slice number.

Values from the series (*u*_*s*_${)}_{s\epsilon N^{*}}$ and (*v*_*s*_${)}_{s\epsilon N^{*}}$ are rounded to the nearest integer so that they correspond to real slice numbers.

The giRAff method is successively executed for each slice *s*, solely on a range of template slices *B*_[us;*vs*]_ ⊂ *B* defined by the two series. This range contains the same number of slices *d*_*t*_/*e*_*t*_ rounded off to the nearest unit. This amounts to determining the *z-position* of the first studied slice from the mutualization of the similarity information *S*_*w*_ of all the slices in the multi-slices set. Once this *z-position* has been estimated in a common manner, it is propagated to the other slices of the series to determine their respective *z-positions*. The position of the other slices is deducted by adding the distance *d*_*t*_ in the template corresponding to the distance *d*_*r*_, which separates the slices from each other in the experimental volume. Assuming *z*_*m*_ is the *z-position* estimated by combining different similarity information in the multi-slices case, as with the classical giRAff method, a calculation of the maximum similarity is then performed to determine the desired position *z*_*m*_:


(11) $$\begin{array}{l} z_{m}\left(E,B\right)=\underset{T_{a}\in B_{\left[u_{s};v_{s}\right]}}{argmax}\left(\frac{1}{n}\overset{n}{\underset{s=1}{\sum }}\pi_{s}S_{w}\left(I_{s},B_{\left[u_{s};v_{s}\right]},{\hat{\theta }{\;}_{\text{RIG}}}_{s},{\hat{\theta }{\;}_{\text{AFF}}}_{s}\right)\right) \end{array}$$

with *S*_*w*_ being a list containing the averaged NMI values for rigid and affine registration (see Equation 3), *E* a multi-slices ensemble, and π_*s*_ the contribution rate for each slice *s* (π_*s*_ = 1/*n* by default, giving an equal contribution for each slice).

Assume giRAff_m_ is the extension of the giRAff method to a multi-slices study, which is defined as:


(12) $$\begin{array}{l} \text{g}iRAff_{m}\left(E,B\right)=\left(z_{s},{\hat{\theta }{\;}_{\text{RIG}}}_{s},{\hat{\theta }{\;}_{\text{AFF}}}_{s}\right) \end{array}$$

For each slice *I*_*s*_ from *E*, a *z*_*s*_ position (deduced from *z*_*m*_) as well as rigid and affine transformations ${\hat{\theta }}_{\text{RIGs}}$ and ${\hat{\theta }}_{\text{AFFs}}$ are determined, which allows the identified label slice *L*_â_ to be mapped onto the experimental single slice *I*_*s*_. The contribution of each slice *I*_*s*_ can be adjusted toward the weight π_*s*_. For example, if a slice *I*_*s*_ has many artifacts that might compromise the registration with the template slices, it is possible to manually adjust its influence by decreasing its contribution π_*s*_ or even remove it from the *z*_*m*_ estimation (π_*s*_ = 0).

The numbers *z*_*s*_ = *t*_*s*_ of each of the slices from the multi-slices study from *E* can directly be calculated from *z*_*m*_:


(13) $$\begin{array}{l} z_{s}=z_{m}+sd_{t}=\hat{t_{1}}+s\frac{e_{t}}{\gamma e_{r}}d_{r} \end{array}$$

with $\hat{t_{s}}$ rounded to correspond to real slice numbers (integers). An affine transformation ${\hat{\theta }}_{\text{AFF}s}$ is associated with each position *z*_*s*_.

#### 2.2.4 giRAffMapper: a generic pipeline

**Figure 4:**
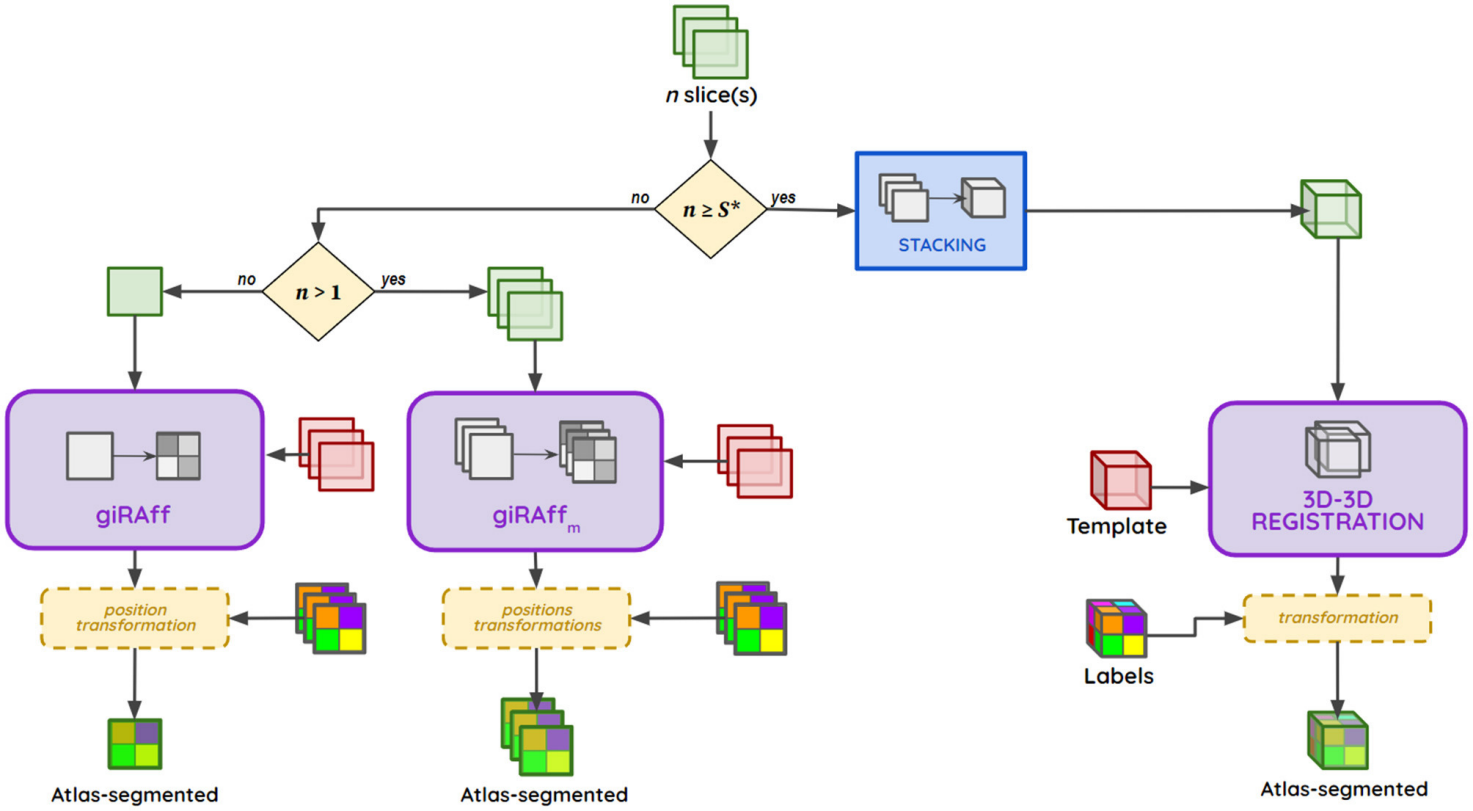
giRAffMapper generic pipeline performing the automated atlas segmentation of any number *n* of histological slices using the giRAff method as well as its extension giRAff_m_. S* represents the needed and required number of slices to reconstruct a 3D brain.

The generic pipeline giRAffMapper automatically performs the atlas segmentation of any number of slices corresponding to any histological experimental protocol. Let S^*^ be the needed and required number of slices to reconstruct a 3D brain. Whether it is for the analysis of a single slice (*n* = 1), for several slices in the analysis of a particular anatomical region (1 < *n* < S^*^), or for a large enough number of slices to perform a 3D reconstruction of the brain (*n* ≥ S^*^), the giRAffMapper generic pipeline automatically processes any histological brain slice protocols (Figure 4).

#### 2.2.5 Validation of the method

##### 2.2.5.1 Metrics of validation

As our aim is to achieve an atlas segmentation as accurate as that of experts, we took the quantitative results of a neuroanatomist's evaluation as a reference. We asked an expert to identify the right number (*z-position*) of the template slice being the most similar to each experimental considered slice *I*_*r*_, the so-called *Expert Rating* for the *z-position* (*ER*_*z*_) (see Supplementary material S1). This made it possible to define the deviation of the *z-position* Δ_*sn*_ between *ER*_*z*_ and the *z-position* estimated by giRAff:


(14) $$\begin{array}{l} \Delta_{sn}\left(I_{r},B\right)=|ER_{z}\left(I_{r},B\right)-z\left(I_{r},B\right)| \end{array}$$

The final purpose being the segmentation of anatomical regions, we also calculated dice scores (Dice, 1945) between the manual segmentation from an expert on the experimental considered slice and the one resulting from the identified and registered template slice by the giRAff method. We then compared these obtained dice scores to those calculated after prior identification of the *z-position* by an expert.

##### 2.2.5.2 Realistic histological protocols to perform region-based analysis

We designed realistic region-based histological protocols from mouse whole brain histological datasets with an expert. Six main regions of interest were chosen from different sizes and locations in the brain: cortex, striatum, hippocampus, thalamus, globus pallidus, and substantia nigra. We especially selected them because of their known involvement in neurodegeneration, especially concerning Alzheimer's, Parkinson's, or Huntington's diseases (Dostrovsky et al., 2002; Picconi et al., 2005; Teichmann et al., 2005). For each anatomical region, the protocol includes the identification of the respective slices in which this region starts and ends along the AP axis, as well as the number of slices to be considered and their inter-slice distance, allowing quantitative studies (see Supplementary material S2). To assess the robustness of such an exploratory approach, we tested all possible protocol combinations covering each region and brain considered, given a constant inter-slice distance.

#### 2.2.6 Determination of the rigid-affine weighting *w* for a given imaging modality

To determine the optimal rigid-affine weighting to be applied for a given imaging modality, we evaluated the average Δ_*sn*_ values for each possible weighting, using steps of 0.01, for all the slices from the brains in a given modality. From this evaluation, we estimated an average curve of Δ_*sn*_ as a function of *w*, which gave us an average trend displaying which rigid-affine weighting *w* minimizes deviation Δ_*sn*_ and, therefore, maximizes the accuracy of the method. To get a realistic idea of this trend for conventional histological slices, it is necessary to exclude from the overall estimate brains suffering from too many artifacts (air bubbles, tearing, missing tissue, etc.) that could compromise this evaluation.

### 2.3 Implementation details and source code

Considering the large number of calculations, the pipeline was run using distributed computing on multiple microprocessors using the SomaWorkflow library of BrainVISA software (Laguitton et al., 2011). BrainVISA is an open-source software platform for neuroimaging research, including visualization tools and graphical user interfaces (https://brainvisa.info). This study was conducted on a workstation Ubuntu 16.04; LTS 64-bits; Intel^®^ Xeon^®^ CPU E5-2620 v2 @ 2.10GHz × 24 (24 computing cores); 128 GB of Random Access Memory (RAM), with the support of our Titan2 calculator composed of five DELL R610 bi-processor nodes on Intel^®^ Xeon^®^ CPU X5675 @ 3.07GHz × 12 and 48 Go of RAM, one DELL R610 bi-processor node on Intel^®^ Xeon^®^ CPU X5667 @ 3.07GHz × 8 and 48 Go of RAM, and six DELL R630 bi-processor nodes on Intel^®^ Xeon^®^ CPU E5-2630 v3 @ 2.40GHz × 16 and 128 Go of RAM (representing 328 computing cores overall).

## 3 Results

The giRAff method has the advantage of being exhaustive in exploring all the possible correspondences after linear registration between a single slice under study and the slices from the average template. This exploration is performed in a minimum of time thanks to a distributed implementation. The choice of the registration algorithm as well as the similarity metric was made to suit multimodal studies, and their independence provides robustness in the identification of the right *z-position* for a given single slice.

The giRAff_m_ extension has been specially designed for multi-slices studies, where the RSF is taken into account for an accurate and realistic estimation of the common *z-position* for a given dataset.

All these developments are gathered in a generic pipeline able to automatically segment any number of slices by atlas. The method presents the advantage of being embedded in an easy-to-use software for simple utilization (see Supplementary material S6).

We used two complementary metrics to evaluate the efficiency of the method in its two different aims: its ability to identify the right *z-position* of single histological slices, whatever their number, and its ability to present relatively good atlas segmentation scores after registration.

### 3.1 Single histological slice segmentation by giRAff

#### 3.1.1 Determination of the rigid-affine weighting *w*

**Figure 5:**
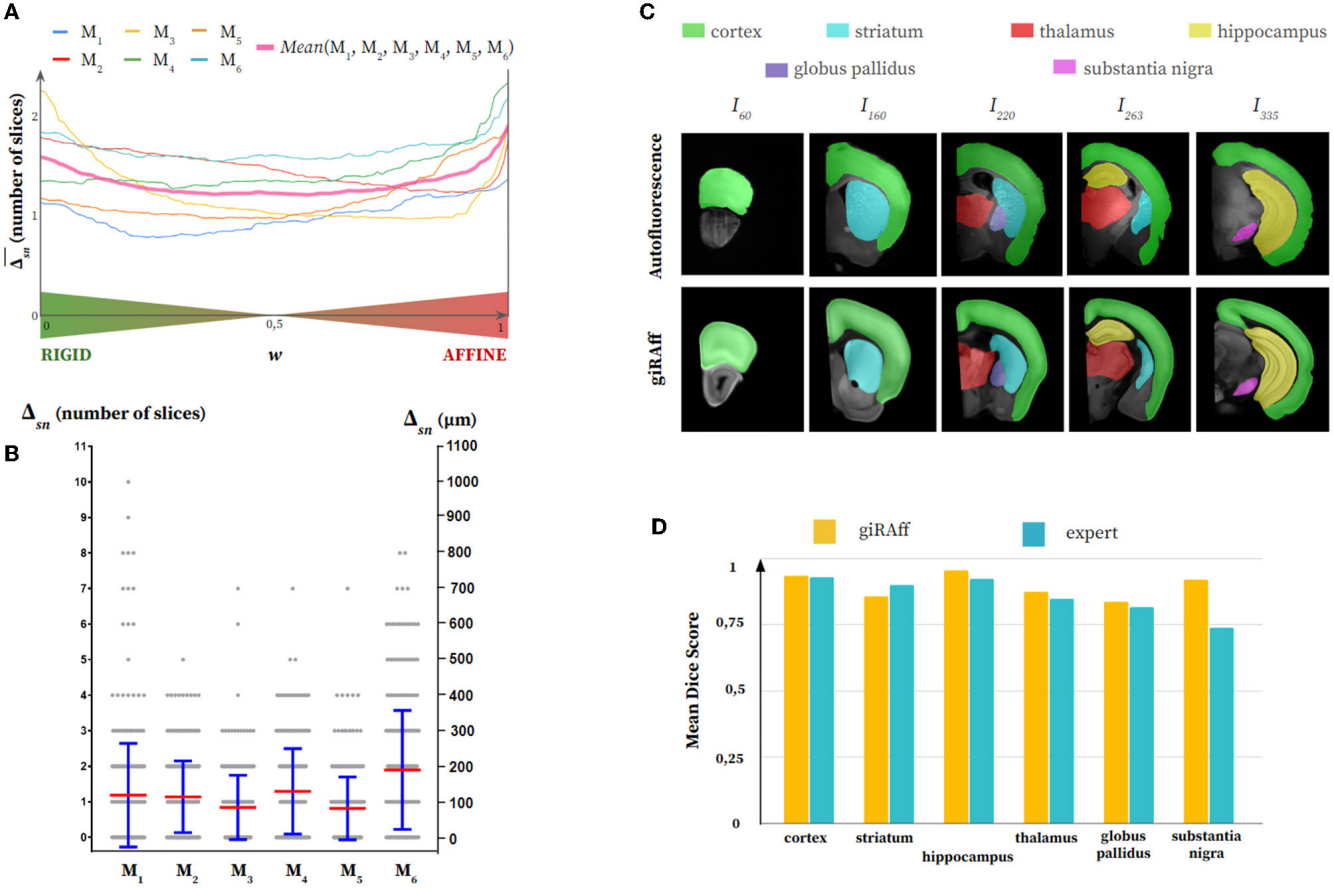
Single slice manual segmentation of the Autofluorescence half mouse brains (M_1_-M_6_): **(A)** Averaged Δ_*sn*_ values after application of the giRAff method for each rigid-affine weighting *w* from 0 to 1 by 1% increments, **(B)** Δ_*sn*_ values (gray) after application of the giRAff method (mean in red and standard deviation in blue) for each single slice, **(C)** segmentation of six anatomical regions of interest of various sizes (cortex, striatum, hippocampus, thalamus, globus pallidus, and substantia nigra) by an expert in five experimental single slices across the brain M_1_ (first row) as well as their corresponding registered template slice identified by the giRAff method (second row), and **(D)** non-weighted mean dice scores (see Supplementary material S4) evaluated on the six anatomical regions for the five slices identified and registered from **(C)** between the giRAff method (yellow) and an expert (cyan).

**Figure 6:**
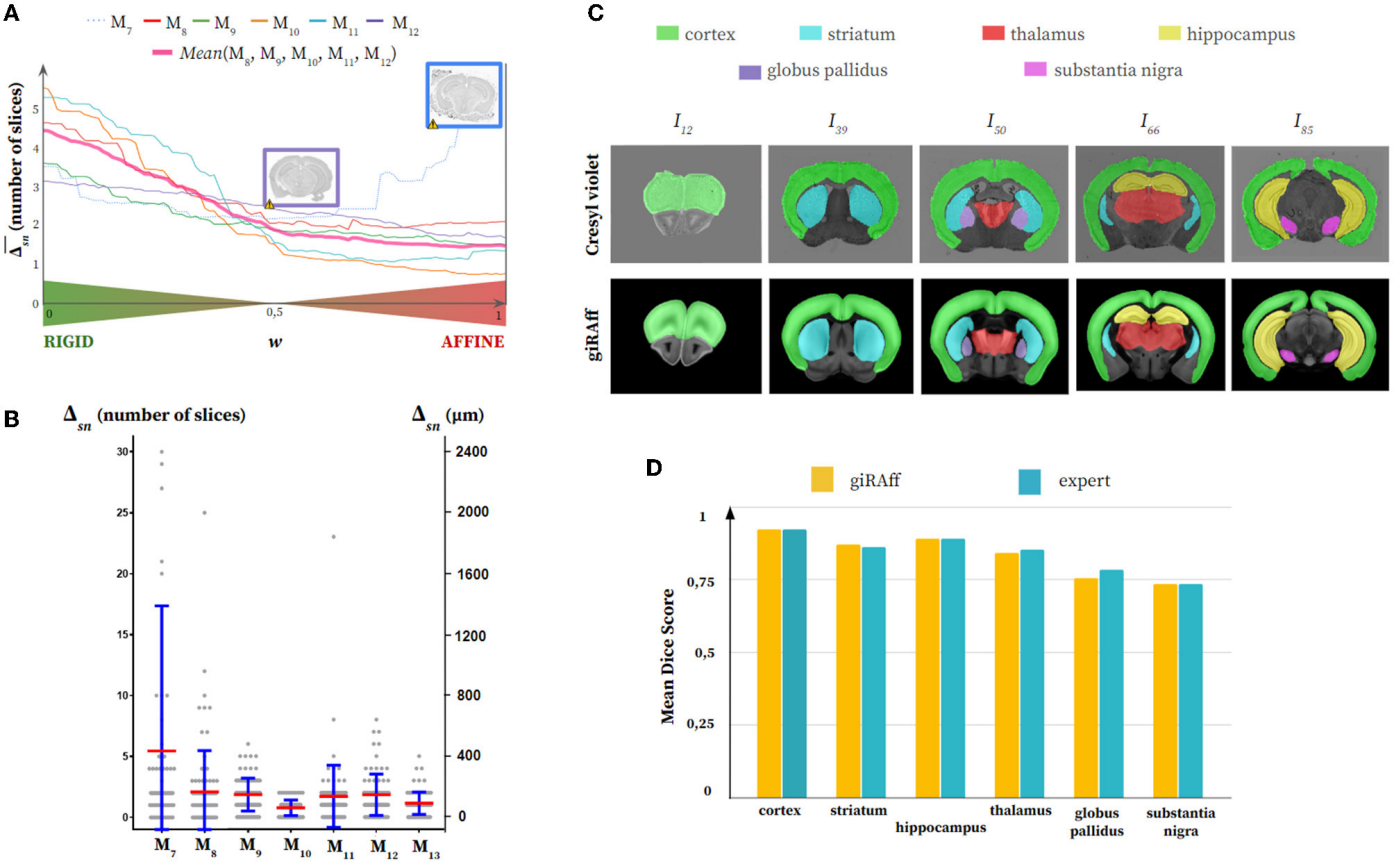
Single slice manual segmentation of the seven cresyl violet mouse brains (M_7_-M_13_): **(A)** Averaged Δ_*sn*_ values after application of the giRAff method for each rigid-affine weighting *w* from 0 to 1 by 1% increments, **(B)** Δ_*sn*_ values (gray) after application of the giRAff method (mean in red and standard deviation in blue) for each single slice, **(C)** segmentation of six anatomical regions of interest of various sizes (cortex, striatum, hippocampus, thalamus, globus pallidus, and substantia nigra) by an expert in five experimental single slices across the brain M_10_ as well as their corresponding registered template slice identified by the giRAff method, and **(D)** non-weighted mean dice scores (see Supplementary material S4) evaluated on the six anatomical regions for the five slices from **(C)** between the giRAff method (yellow) and an expert (cyan).

We first evaluated which rigid-affine weight *w* minimizes the Δ_*sn*_ criterion for each modality: the autofluorescence (Figure 5A) and the cresyl violet (Figure 6A). For the autofluorescence, the trend was clearly not toward a rigid-affine weighting *w* at extremes (0 or 1). No particular weighting appeared to be especially optimal between these extreme values. We therefore chose a rigid-affine weighting *w* = 0.50 for this modality to ensure robustness in the use of the two types of registration and to avoid the extreme weightings, which can be a source of misidentification (high Δ_*sn*_). Concerning cresyl violet, it was necessary to remove data presenting too important artifacts (M_7_), making them non-representative for the evaluation of the global trend of the rigid-affine weighting *w*. In contrast to autofluorescence modality, a clear trend appeared in favor of a weighting *w* = 1 for the cresyl violet, which minimized mean Δ_*sn*_. This means that the NMI resulting from affine registration prevailed for this imaging modality in the estimation of the *z-position* of single slices in comparison to an expert.

#### 3.1.2 Precision and robustness of the method

The giRAff method was applied independently on 2,135 single half-slices (one hemisphere) and 636 whole slices (whole brain) from two modalities from 13 mouse brains. In routine protocols performed in our laboratory, φ and β angles were estimated below 5° (see Supplementary material S3) and were neglected in this study. The deviation Δ_*sn*_ compared to an expert was calculated for every single slice considered from this dataset. The giRAff method was able to identify any single mouse brain slice with an average accuracy of 1.20 ± 1.19 and 2.05 ± 3.05 slices for the autofluorescence and the cresyl violet, respectively (Table 1). This represented an average precision of the *z-position* identification between 120 and 164 μm, respectively.

**Table 1:** Single slices—autofluorescence and cresyl violet.

**Autofluorescence**		**M_1_**	**M_2_**	**M_3_**	**M_4_**	**M_5_**	**M_6_**	**MEAN**	
$\bar{{△}_{sn}}\left(\text{nb of slices and }\mu \text{m}\right)$	μ	1.19	1.14	0.84	1.30	0.82	1.90	1.20	
		119 μm	114 μm	84 μm	130 μm	82 μm	190 μm	120 μm	
	σ	± 1.46	± 1.01	± 0.91	± 1.20	± 0.89	± 1.67	± 1.19	
		± 146 μm	± 101 μm	± 91 μm	± 120 μm	± 89 μm	± 167 μm	± 119 μm	
*M* (nb of slices per brain)		354	379	341	342	362	357	2,135	
**Cresyl violet**		**M**_7_	**M**_8_	**M**_9_	**M**_10_	**M**_11_	**M**_12_	**M**_13_	**MEAN**
$\bar{{△}_{sn}}\left(\text{nb of slices and }\mu \text{m}\right)$	μ	5.40	2.07	1.85	0.76	1.70	1.82	1.13	2.05
		432 μm	166 μm	148 μm	61 μm	136 μm	146 μm	90 μm	164 μm
	σ	± 11.84	± 3.38	± 1.35	± 0.64	± 2.54	± 1.69	± 0.91	± 3.05
		± 947 μm	± 270 μm	± 108 μm	± 51 μm	± 203 μm	± 135 μm	± 73 μm	± 244 μm
*M* (nb of slices per brain)		82	93	95	97	93	85	91	636

Average Δ_*sn*_ scores and standard deviation resulting from the application of the giRAff method compared to expert *z-position* evaluation (*ER*_*z*_), as well as the number of slices *M* per brain on which it has been estimated for the six autofluorescence half mouse brains (M_1_-M_6_) and the seven cresyl violet mouse brains (M_7_-M_13_). The MEAN column presents the non-weighted average Δ_*sn*_ values for all the considered brains.

Concerning the autofluorescence, no high Δ_*sn*_ scores appeared, being mainly narrow around 0 and 200 μm, the largest deviation of 10 slices being obtained only once (M_1_) among the six brains (Figure 5B). If we look qualitatively at the segmentation of the anatomical regions of interest, we notice that their delineation is close to that performed by an expert on the experimental slice (Figure 5C). From the smallest of the regions studied (substantia nigra) to the most elongated (cortex), the segmented shapes were quite close. These results were confirmed quantitatively by the dice scores (Figure 5D; see Supplementary material S4) evaluated on five slices among the brain M_1_, which demonstrated the capacity of the giRAff method to obtain fairly high scores (around 0.90) after identification of the *z-position* for a given experimental slice. More importantly, those dice scores were widely comparable to those of an expert.

Results for cresyl violet appeared to be somewhat less accurate, with Δ_*sn*_ scores being concentrated more between 0 and 300 μm on average, with the exception of M_7_, for which values were significantly higher (Figure 6B). Misidentifications above 10 slices of deviation were also rare. The qualitative analysis of the segmentations showed that the anatomical regions corresponded rather well, with some small differences, in proportion for the substantia nigra or in shape for the striatum (Figure 6C). These small differences had very little impact on the dice score, which remained globally quite high (around 0.85), except for the substantia nigra and the globus pallidus (around 0.75). Similarly and most importantly, dice scores showed that segmentation results using the giRAff method on these five slices were still widely comparable to those of an expert (Figure 6D; see Supplementary material S4).

All in all, no particular difference in the accuracy of the giRAff method was noticed in identifying the *z-position* of slices from a brain including pathological lesions (M_13_) compared to other brains (M_7_-M_12_): average Δ_*sn*_ and standard deviation (1.13 ± 0.91 slices) of *M*_13_ were significantly inferior to the mean evaluation on the whole cresyl violet dataset (2.05 ± 3.05 slices).

### 3.2 Multi-slices histological segmentation by giRAff_m_

Based on the rigid-affine weighting empirically determined for each of the two modalities, the giRAff_m_ extension was applied on multi-slices datasets based on routine histological sectioning protocols. Those protocols were designed by experts to correspond to studies of particular anatomical regions of different sizes in the coronal incidence: cortex, striatum, thalamus, hippocampus, globus pallidus, and substantia nigra (see Supplementary material S2). These conventional protocols involved a number of slices and an inter-slice distance, with the first slice of a given region being shifted at each iteration so that the entirety of the slices constituting each region were tested. The deviation Δ_*sn*_ was calculated for each slice included in every multi-slices case, and the result was averaged per anatomical region studied.

The deviation Δ_*sn*_ was estimated in three different contexts: (1) using the giRAff method considering each slice as single (same case as in Section 3.1 focused on the slices including each anatomical region considered), (2) using the giRAff_m_ extension considering multi-slices protocols and an RSF γ_M*i*_ evaluated for each brain thanks to the *ER*_*z*_, and (3) using the giRAff_m_ extension considering multi-slices protocols and an averaged RSF γ_m_ evaluated for a given protocol and imaging modality (see Supplementary material S5).

**Figure 7:**
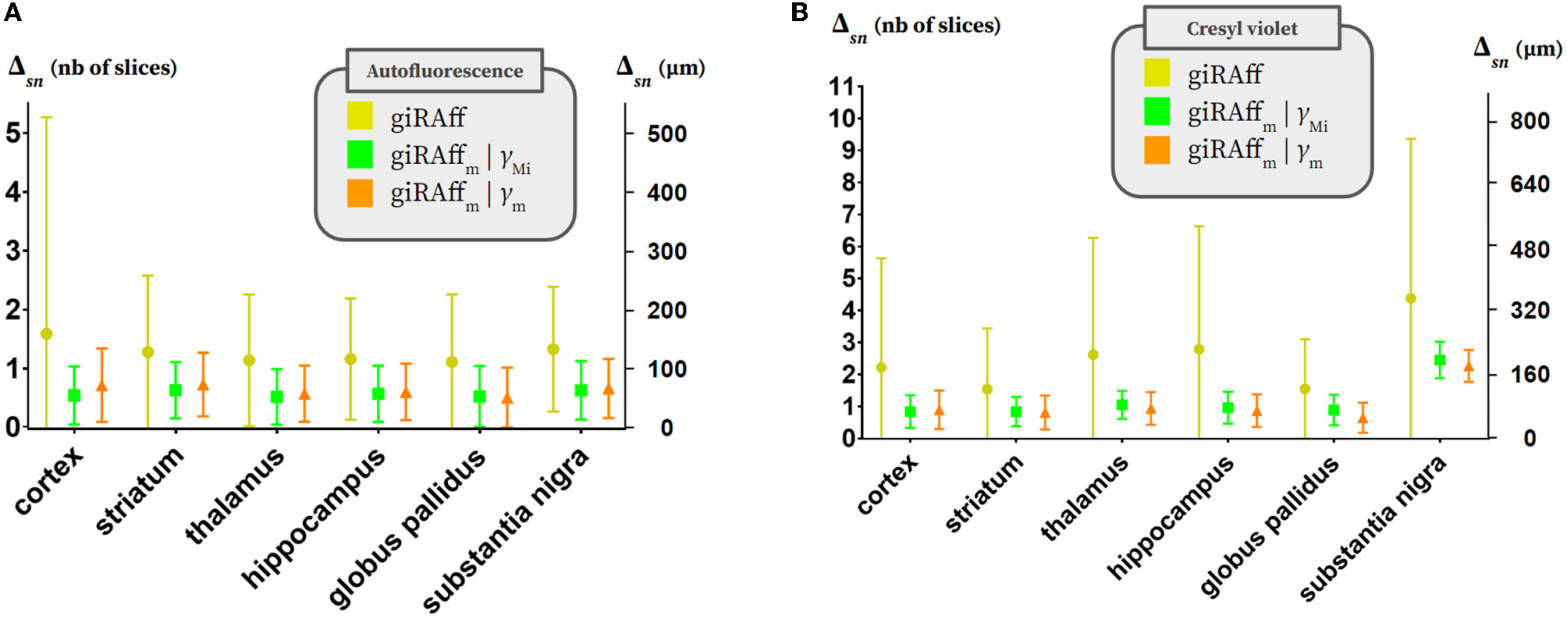
Multi-slices. Averaged Δ_*sn*_ values and standard deviation per considered anatomical region (cortex, striatum, hippocampus, thalamus, globus pallidus, and substantia nigra) in three different contexts: using the giRAff method considering each slice as single (yellow), using the giRAff_m_ extension considering multi-slices and RSF γ_M*i*_ evaluated for each brain thanks to the *ER*_*z*_ (green), and using the giRAff_m_ extension considering multi-slices and an averaged RSF γ_m_ evaluated for a given protocol and imaging modality (orange) for **(A)** the six autofluorescence brains and **(B)** the seven cresyl violet brains.

Concerning the autofluorescence, first, the multi-slices approach significantly reduced the average deviation Δ_*sn*_ and its dispersion, in general: Δ_*sn*_ criterion underwent a reduction between 55 and 105 μm and the standard deviation between 53 and 87% depending on the region, on average (Table 2; Figure 7A). The case of considering the RSF γ_M*i*_ specific to each volume M_*i*_ (*i* ranging from 1 to 6) presented smaller deviations Δ_*sn*_ than the case of an average RSF γ_m_ (increase of the order of 8%). Depending on the experimental conditions that were applied to each volume, considering γ_M*i*_ specific to each of them made it possible to obtain better accuracy in the detection of the *z*_*m*_ position. Estimating an accurate value of this RSF γ increased the precision of detecting the right position *z*_*m*_ by the giRAff_m_ extension. On average, over all regions, the accuracy of *z*_*m*_ position detection in the multi-slices case by the giRAff_m_ extension was equal to 57 ± 49 μm with γ_M*i*_ and 63 ± 52 μm with γ_m_ for the autofluorescence.

**Table 2:** Multi-slices—autofluorescence and cresyl violet.

**Autofluorescence** Δ***_*****sn*****_*** **(nb of slices and** μ**m)**		**Cortex**	**Striatum**	**Thalamus**	**Hippocampus**	**Globus pallidus**	**Substantia nigra**	**MEAN**
giRAff	μ	1.59	1.28	1.14	1.16	1.11	1.33	**1.27**
		159 μm	128 μm	114 μm	116 μm	111 μm	133 μm	**127** **μ*m***
	σ	± 3.68	± 1.30	± 1.12	± 1.03	± 1.15	± 1.06	**±** **1.56**
		± 368 μm	± 130 μm	± 112 μm	± 103 μm	± 115 μm	± 106 μm	**±** **156** **μ*m***
giRAff_m_ | γ_M*i*_	μ	0.54	0.63	0.52	0.57	0.53	0.63	**0.57**
		54 μm	63 μm	52 μm	57 μm	53 μm	63 μm	**57** **μ*m***
	σ	± 0.49	± 0.48	± 0.47	± 0.48	± 0.52	± 0.50	**±** **0.49**
		± 49 μm	± 48 μm	± 47 μm	± 48 μm	± 52 μm	± 50 μm	**±** **49** **μ*m***
giRAff_m_ | γ_m_	μ	0.72	0.73	0.57	0.60	0.51	0.66	**0.63**
		72 μm	73 μm	57 μm	60 μm	51 μm	66 μm	**63** **μ*m***
	σ	± 0.62	± 0.54	± 0.48	± 0.48	± 0.51	± 0.50	**±** **0.52**
		± 62 μm	± 54 μm	± 48 μm	± 48 μm	± 51 μm	± 50 μm	**±** **52** **μ*m***
**Cresyl violet** Δ*_*sn*_* **(nb of slices and** μ**m)**		**Cortex**	**Striatum**	**Thalamus**	**Hippocampus**	**Globus pallidus**	**Substantia nigra**	**MEAN**
giRAff	μ	2.22	1.54	2.62	2.79	1.55	4.38	**2.31**
		178 μm	123 μm	210 μm	223 μm	124 μm	350 μm	**286** **μ*m***
	σ	± 3.41	± 1.90	± 3.65	± 3.84	± 1.55	± 4.99	**±** **3.17**
		± 273 μm	± 152 μm	± 292 μm	± 307 μm	± 124 μm	± 399 μm	**±** **254** **μ*m***
giRAff_m_ | γ_M*i*_	μ	0.84	0.84	1.05	0.96	0.89	2.45	**0.98**
		67 μm	67 μm	84 μm	77 μm	71 μm	196 μm	**78** **μ*m***
	σ	± 0.51	± 0.46	± 0.44	± 0.50	± 0.48	± 0.57	**±** **0.49**
		± 41 μm	± 37 μm	± 35 μm	± 40 μm	± 38 μm	± 46 μm	**±** **39** **μ*m***
giRAff_m_ | γ_m_	μ	0.90	0.81	0.94	0.87	0.65	2.27	**0.94**
		72 μm	65 μm	75 μm	70 μm	52 μm	182 μm	**75** **μ*m***
	σ	± 0.60	± 0.53	± 0.51	± 0.51	± 0.47	± 0.50	**±** **0.54**
		± 48 μm	± 42 μm	± 41 μm	± 41 μm	± 38 μm	± 40 μm	**±** **43** **μ*m***

Non-weighted average Δ_*sn*_ scores (deviation in number of slices) and standard deviation calculated on six anatomical regions of interest of various sizes (cortex, striatum, hippocampus, thalamus, globus pallidus, and substantia nigra) using realistic conventional histological protocols for the six autofluorescence brains (M_1_-M_6_) and the seven cresyl brains (M_7_-M_13_) in three different contexts: (1) using the giRAff method considering each slice as single, (2) using the giRAff_*m*_ extension considering multi-slices and RSF γ_M*i*_ evaluated for each brain thanks to the *ER*_*z*_, and (3) using the giRAff_*m*_ extension considering multi-slices and an averaged RSF γ_*m*_ evaluated for a given protocol and imaging modality (autofluorescence and cresyl violet).

Regarding the cresyl violet, second, the multi-slices approach strongly decreased the average deviation Δ_*sn*_ and its dispersion in general: Δ_*sn*_ criterion underwent a reduction between 53 and 169 μm, and the standard deviation between 69 and 90% depending on the region, on average (Table 2; Figure 7B). The use of the giRAff_m_ extension in the multi-slices case significantly improved the overall detection accuracy of the *z*_*m*_ position in this modality. In contrast to what was observed for the autofluorescence data, the γ_m_ case presented better results (Δ_*sn*_ decreased by 4% on average over all regions) than for the consideration of the respective γ_Mi_. Only the cortex region showed Δ_*sn*_(γ_M*i*_) > Δ_*sn*_(γ_m_) by 7%. For the other regions, considering γ_m_ rather than γ_M*i*_ improved the detection of the correct *z-position* by 4% (striatum) to 27% (globus pallidus). On average, over all regions, the accuracy of *z*_*m*_ position detection in the multi-slices case by the giRAff_m_ method was 94 ± 54 μm with γ_m_. Whatever the case considered, the substantia nigra was the only region with high deviations: the accuracy Δ_*sn*_ was 180 ± 40 μm while it was always <80 μm for all other regions. Atlas segmentation of small anatomical regions was more challenging than for large regions, both for experts and for the proposed method.

A gain in accuracy was clearly observed when using the giRAff_m_ extension compared to the giRAff method for the same slices considered independently: with a few exceptions, Δ_*sn*_ was brought down between 0 and 100 μm on average, whatever the modality and the region.

For one single slice, the giRAff method proposed an automated atlas segmentation in about 1 min using Titan2 infrastructure.

### 3.3 Cross-talk between giRAff and giRAff_m_

**Figure 8:**
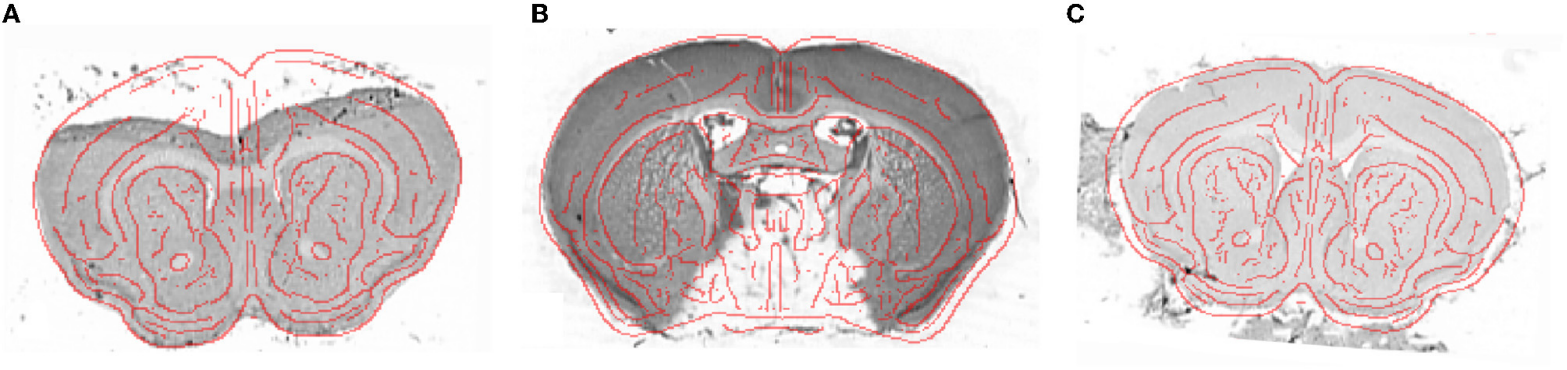
Example of histological single slices (cresyl violet) presenting histological artifacts: **(A)** tissue folding (M_11_), **(B)** missing tissue (M_8_), and **(C)** external noise (M_7_), with the superposition of the red boundaries of the template slice, obtained using a Deriche filter (Deriche, 1990), at the *z-position* identified by the giRAff_m_ extension.

Several single slices from cresyl violet mouse brains (M_7_-M_13_) suffered from histological artifacts. In most cases, the presence of a considerable artifact prevents segmentation of the entire histological slice. Such a slice is often discarded, or its segmentation is carried out manually if the damaged part does not concern the tissue of interest. Despite some considerable artifacts, the giRAff_m_ extension still allows for identification of the correct *z-position* and segment the rest of the slice correctly. Some examples including such artifacts (tissue folding, missing tissue, and external noise) are presented in Figure 8.

## 4 Discussion

In this study, we proposed a method to automatically segment one or a set of single slices using a 3D digital atlas. The giRAff method, based on linear registration tools and on the NMI as a similarity metric, showed its ability to deal with any number of slices, adapting to very different standard histological protocols (3D fluorescence and 2D brightfield imaging). We demonstrated the robustness and the efficiency of the method by applying it on two different datasets: autofluorescence data, which was not affected by cutting artifacts, and histological slices from routine experimental protocols. It was indeed able to identify, depending on the protocol considered, the *z-position* of one or more single slice(s) with an accuracy of the order of one slice within the atlas template. This amounted to an identification deviation of less than about 100 μm on average, with dice scores comparable to those obtained by an expert. The method also showed its ability to deal with slices suffering from histological artifacts using the multi-slices approach.

The method was based on a balanced use of the similarity information evaluated after rigid and affine registration in an exploratory approach. In this context, the rigid-affine weighting *w* was of crucial importance as it allowed to adjust the use of NMI information to take advantage of the benefits from each type of registration. Indeed, in the exploratory approach we proposed, the two types of registration can be complementary. Rigid registration is often rough and avoids the identification of a particular slice that is the closest to the single slice considered, whereas affine registration makes the difference in improving tissue registration thanks to a greater number of degrees of freedom (shearing and scaling). On the contrary, affine registration could make slices correspond to each other with an inappropriate superposition of tissues forced by large deformations, whereas rigid registration does not allow such modifications, limits the deformations, and permits the differentiation of these slices. The use of a weighted proportion of the similarity information created a robust study framework for their comparison in an exploratory context. This represents a useful parameter to tune according to the amplitudes of the deformations considered or according to the biological protocol used. For the two modalities tested in this study, the trend was toward either 0.5 or 1. What we would suggest for users is to consider one or the other of the rigid-affine weighting given in the manuscript by default for their own data according to the imaging modality chosen. In the case of another specific imaging modality or for any doubt on the rigid-affine weighing chosen, the operator could easily test adjusting it from 0.5 to 1 or from 1 to 0.5. If this improves the result in their opinion for their own dataset, they should obviously reuse it by default for the next iterations with other data produced in the same modality. Initialization of the registration by centering the slices on each other was therefore a mandatory step in this gradual pipeline. Even if this centering process was presented as being manually performed in this study, it would be possible to readily add a simple algorithm to perform this task in an automated manner. Maximizing the overlap of the binarized tissue surface could be used, for example, to improve the method in the future.

The giRAff method was inspired by the operating mode an expert uses when manually identifying the position of a single slice: neuroanatomists flip atlas pages and try to match the shapes of certain anatomical regions in an exploratory way, as well as qualitatively estimate the similarity in a visual manner. Our pipeline does the same using linear registration and NMI. Although NMI has shown its robustness in various multimodal brain applications, its efficiency remains discussed within the scientific community (Zheng, 2006; Xiong et al., 2018; Song et al., 2020). This similarity metric is known to have non-significative values in absolute: comparing two objects whose nature does not have anything in common can even result in a significantly high NMI score (Rohlfing, 2011). The use of NMI was solely relative in our pipeline, considering its score on any template slice in comparison to each other. This information was never used in an absolute manner, and the nature of the objects being compared was the same, thus avoiding this limitation. The NMI was not used as a similarity metric to estimate registration but only to objectively evaluate the quality of the slice-to-slice correspondence after registration.

In the dataset we used, we purposely selected uncut 3D coherent histological brain volumes (autofluorescence of a cleared brain acquired with a light-sheet microscope), which was considered as a succession of 2D virtual slices. In such a way, it was possible to test different data processing approaches with artifact-free tissue. This could be one of the reasons why the precision of the *z-position* detection was better for the autofluorescence (lower Δ_*sn*_) than for the cresyl violet. We first opted for this favorable context to make a proof of concept, taking autofluorescence as a kind of ideal case (Piluso et al., 2021a). Then, we confronted with “real life” histological preclinical routine protocols (digitized Nissl/cresyl violet-stained brain sections), based on our robust and adjustable pipeline.

When using the giRAff method for a given individual slice, its *z-position* is estimated only once. This estimate may suffer from deviations that could be due to the presence of artifacts in the slices, by poor quality registration, or by a relative similarity value that is not significant enough. In the giRAff_m_ approach, the joint estimation of the position *z*_*m*_ from a set of slices provided a statistical quantity of estimates sufficient to significantly reduce the deviation Δ_*sn*_ and its dispersion in general. This improvement was based on the assumption that a large majority of the slices had little or no artifact, and that the registration and similarity metric were robust enough to accurately estimate the *z-position* of such slices in the dataset. As a result, for one or several slice(s) suffering from artifacts, representing a minor proportion of a given dataset, giRAff_m_ provided better *z-position* identification results than giRAff.

On average, for a multi-slices dataset, the *z-position* of single slices was detected with a precision of one slice in the atlas (~ 100 μm). This deviation is comparable to the one that experts could make on such a dataset, as long as one single slice considered does not perfectly match one given slice in the template. Indeed, because of its slice thickness and its exact location on the AP axis, as well as possible tilting angles, experts sometimes hesitate between two adjacent slices from the template to identify the right *z-position* of an experimental single slice. Therefore, they are constrained to make an arbitrary choice, assuming that the position they have identified is only accurate within one slice (100 μm).

Some regions with little pixel support, such as substantia nigra, presented poor dice score results compared to other bigger regions with larger pixel supports. In the linear registration algorithm used, few degrees of liberty were allowed to try to optimize a global transformation at a whole-image scale. This obviously tended to maximize the overlap between regions, including larger pixel support at the expense of other smaller regions including significantly fewer voxels. In such regions, a difference of one single voxel was far more significant than in other regions. Using non-linear registration after estimating the *z-position* could significantly increase the overlap between such small regions and then significantly increase their dice score.

Concerning the cresyl violet data, the M_7_ brain showed higher Δ_*sn*_ scores (larger deviation) than the other brains without using the multi-slices extension giRAff_m_, confirmed by the presence of artifacts due to the histological and digitization protocols (bubbles, added tissue fragments, and external noise). This is a typical example of artifacts that can occur during a conventional histological protocol. Automatically segmenting histological slices with significant artifacts has always been a challenging task for the scientific community (Agarwal et al., 2016). Most of the time, automatic atlas segmentation of these slices is basically impossible. Our proposed giRAff_m_ extension has the advantage of optimizing *z-position* detection on a set *E* of multiple single slices, and thus could be able to identify and segment such slices including artifacts. Results were pooled to obtain the best *z*_*m*_ position estimated for all the slices. Thus, for a set of slices from the same brain, including slices with important artifacts, it was then possible to decrease their rate of contribution π_*s*_ (until 0) in the global estimation of the *z*_*m*_ position, but yet achieve their automatic segmentation reliably. Considering a majority of good quality slices selected from *E* and a robust regression (significantly high coefficient of determination, typically above 0.97), the giRAff_m_ extension can propose an automated atlas segmentation corresponding for any other slice suffering from those artifacts from the same brain in a robust way, especially without taking them into account in the global estimation of the position *z*_*m*_. If the rate of contribution π_*s*_ was presented as a subjective parameter to add manually as input information within the multi-slices pipeline, further improvements could lead to the use of image processing algorithms able to automatically detect artifacts within histological slices (Agarwal et al., 2016). This would lead to an automated setup of the rate of contribution π_*s*_ for each single slice as a consequence. Moreover, using the multi-slices giRAff_m_ extension allowed for automated estimation of the RSF γ between the data considered. This reinforced the fact the method we proposed is versatile, robust, and adaptable to many types of protocols or histological brain data.

Considering a multi-slices dataset, we focused on a constant inter-slice distance between single slices under study in this article. But in practice, this distance could be heterogeneous. The principle of the multi-slices extension giRAff_m_ for the analysis of such slices would be exactly the same; the different inter-slice distances can be given as input information within the giRAff_m_ pipeline.

In conventional histological protocols, tilting angles may occur when slicing the 3D organ. A non-zero φ angle around the IS axis can generate anatomical differences between the left and right side of the slice, which are easy for an expert to identify due to the brain symmetry with respect to the interhemispheric plane. However, it is more challenging to identify a non-zero β angle around the LR axis that will generate differences between the top and bottom of the slice. This angle is most often observed as non-zero, and neurobiologists then have to deal with neighboring slices to perform the segmentation manually. Thanks to a rigid 3D-3D registration between each considered brain and the template volume, it was possible to estimate these tilting angles around the IS and LR axis, and they are of low amplitude (<5°, see Supplementary material S3), hence our focus on the *z-position* determination. Considering those realistic tilting angles of low amplitude, the accuracy of the giRAff method nevertheless made it possible to preserve automatic segmentations for which dice scores are still comparable to those of an expert. Indeed, as protocols for acquiring those brains may be representative of standard protocols in conventional brain histology performed in the coronal incidence, we assume that tilting angles rarely exceed an amplitude of 5° with modern equipment and in a similar study framework. If this angulation generates genuine anatomical differences compared to data without angulation, the method we proposed made it possible to compensate for this drawback. Indeed, we chose to process data produced in routine histological protocols in this article, i.e., including real tilting angles caused by the cutting process. Histological data presented in this article included their native tilting angles. As the giRAff method detected the *z-position* of the single slice with high accuracy, its anatomical environment was well identified (basically in the thickness range of about 200 μm). Following this location, registration ensured the best matching of the tissue between the single slice considered and the template slice identified, as it would have been done in the case of considering the respective adjacent slices of its direct neighborhood. In the coronal incidence and with a slice thickness of about a hundred micrometers, anatomical variations are small from one slice to the next adjacent one. The template data are smooth, and very few discontinuities appear when examining the slices one after the other along the AP axis. More specifically, a tilting angle would generate small anatomical differences between the right and left of the slice for an angle φ around the IS axis and between the top and bottom of the slice for an angle β around the LR axis compared to the template data. In practice, using linear registration would basically correct most of those segmentation errors because the presence and location of anatomical regions are almost the same from one slice *n* to its *n*-1 and *n*+1 (or more) neighbors. Indeed, anatomical differences generated by a tilting angle cause linear deformations along one, two, or both axes (IS and LR in the coronal incidence), which affine registration can compensate with shearing. This was confirmed by dice scores calculated, which were widely comparable to those of an expert in the end. The only necessary condition is that the *z-position* of the single slice considered must be accurately estimated, typically with a deviation less or equal to one slice in the template, to avoid too large anatomical difference between slices considered. It is just a matter of comparing data which are comparable, i.e., extracted, cut, and digitized within a rigorous, consistent, and realistic study framework. If neurobiologists are asked to cut coronal mouse brain slices using a microtome, it is reasonable to believe that their skills will enable them to obtain tilting angles below 5° as observed in the data presented in this article. Visual quality control and steel matrices could also be used for this purpose.

The method we proposed was based on linear registration in a pipeline with an increasing number of degrees of freedom. The use of non-linear registration could compromise the identification of the correct *z-position* of a given single slice. Indeed, too many degrees of freedom would excessively distort all template slices to match, in an inappropriate way, the single slice under consideration. It would then be challenging to distinguish which was the most similar. In contrast, the use of non-linear 2D-2D registration between the single slice and the template slice identified at the *z-position* at the end of the giRAff pipeline would certainly enable the segmentation results to be refined. This could be useful for the analysis of small regions, for example. This constitutes one of the further improvements the method could benefit from. Moreover, the lack of ground truth will make the task even harder.

A benchmark between the different methods of segmenting single slices should be carried out to identify which could give the best results according to the experimental data under study. Such a benchmark should accurately compare all the methods using a dedicated common dataset as well as an appropriate metric to evaluate their respective performance. This comparison is too vast to be presented exhaustively and precisely in this paper and could be the scope of another study. Indeed, each method has its own particular way of working, and its results may be of a different nature, making them difficult to benchmark. Nevertheless, we wanted to briefly test whether our method offered competitive results compared with those provided by the most recent state-of-the-art method. A quick comparison was led on two independent single coronal Nissl-stained slices between the latest fully automated method from the state-of-the-art (DeepSlice from Carey et al., 2023) and our giRAff method. We estimated NMI similarity metric after applying both methods in the same conditions. Those unitary tests showed that similarity between the resulting slices from our method outperformed DeepSlice by about 20%, while requiring a longer processing time (<30 s for DeepSlice and about 1 min for giRAff, estimated per slice). Looking at the anatomy in the identified template slices, the *z-position* determined for both methods was very close, if not equal. Only some slight registration differences were observed, where the registration algorithm used in giRAff provided the best results according to the NMI criterion. These were very preliminary unitary tests, hence the need for this benchmark to be fully explored in future.

The giRAff method was developed to be fully automatic and embedded in an easy-to-use interface with very few input parameters so that it can be easily used by a non-expert. Optional parameters can be adjusted if the user wants to contribute with their own knowledge, such as the selection of the region(s) of interest studied. This information will reduce the number of adjacent template slices to consider in estimating the *z-position* of a single slice. Only template slices including this or those anatomical region(s) will be pre-selected, thus decreasing the computation time.

In automatic mode, the method segments a single slice in 1 min on a high-performance computing infrastructure. The result benefits not only from the six regions we focused on but from all the subregions defined in the ABA reference. This is comparable to the time it may take an expert to identify the correct *z-position* of a single slice within the atlas template. For the same processing time, the giRAff method additionally provided direct atlas segmentation of the single slice. Moreover, no knowledge of brain anatomy or even in coding was required to use the method. Its interface and the few input parameters required by our pipeline make it usable by anyone with full autonomy. Even without supercomputer infrastructure, using about 20 computing cores from a workstation, for example, the method for one single slice worked in a reasonable time of about 15 min.

First, preliminary results as well as complementary studies on a brain suffering from pathological lesions showed encouraging results for the method to be able to handle such data in the context of dedicated protocols. This opens the door for automated segmentation of slices from pathological mouse models, whether neurodegenerative or other diseases, as long as data did not suffer from too large anatomical alterations. Similarly, the use of this pipeline can be extended to other rodents, such as rat for instance, or even in other modalities, such as magnetic resonance imaging. Promising results have been obtained on this modality (Piluso et al., 2021b), and future work aims at validating the use of the method in such cases. In addition, the use of this method will indirectly allow better targeting of conventional histology protocols to reduce the amount of brain data to be used in a study.

## 5 Conclusion

The wide variety of existing histological protocols as well as the great numbers of anatomical structures in the mouse brain makes the analysis of histological slices quite tedious and complex. In conventional preclinical histology for the analysis of the mouse brain, it is rare to have enough slices to reconstruct the brain in 3D and, sometimes, working on 3D data is not a prerequisite. It is possible to study only one single slice within the brain, but this is also unusual. In contrast, many protocols are based on a fairly large number of slices to perform quantitative studies on particular anatomical regions or around a specific pathological lesion, for example, still precluding 3D reconstruction. Whatever the case, the generic giRAffMapper pipeline was optimized to accommodate most protocols involving any number of single slices. We showed that our method was able to automatically identify the position of single slices within a mouse brain atlas with less than one slice deviation on average and in 1 min for one slice. Atlas segmentations were comparable to those of an expert. The giRAff method does not need any 3D brain volume reconstruction; it is versatile, generic, user-friendly, and requires few input parameters. In future, we aim to take into account real slice angles and use non-linear registration tools to further refine the segmentation of anatomical regions from increasingly precise atlases. This study paves the way for automated atlas segmentation through a simplified interface of any histological mouse slice, half- or whole-brain slice, for pathological models, for different modalities and possibly for different species. This is done in a fully automated way and does not require any particular knowledge of the study involved, nor in neuroanatomy in general, nor even in coding, to be able to use it. This significantly widens the scope of use of such anatomical detailed atlases within the scientific community for a complex task that usually had to be performed only by experts.

## Data availability statement

The original contributions presented in the study are included in the article/Supplementary material, further inquiries can be directed to the corresponding author.

## Ethics statement

The animal study was approved by dedicated Institutional Animal Care and Ethics Committees, where the experimental procedures involving animal models described in this paper come from already published papers (Renier et al., 2016; Vandenberghe et al., 2016). The study was conducted in accordance with the local legislation and institutional requirements.

## Author contributions

All authors listed have made a substantial, direct, and intellectual contribution to the work and approved it for publication.
