# Supplementary material for: giRAff: an automated atlas segmentation tool adapted to single histological slices
Source: Front Neurosci. 2024 Jan 11;17:1230814. doi: 10.3389/fnins.2023.1230814 (PMC10808556; doi:10.3389/fnins.2023.1230814)
Supplement: Supplementary file 1 [file Data_Sheet_1.pdf]

SUPPLEMENTARY MATERIAL

S0. Biological and acquisition protocols

Autofluorescence

For this first dataset, the full details for the biological and acquisition protocols are available in Renier et al., 2016.

Sample clearing

Samples were dehydrated in 20% methanol (in ddH2O) for 1h, 40% methanol / H2O for 1h, 60% methanol / H2O for 1h, 80% methanol / H2O for 1h, and 100% Methanol for 1h twice. Samples were incubated overnight in 1 volume of Methanol / 2 Volumes of Dichloromethane (DCM, Sigma 270997-12X100ML) until they sank at the bottom of the vial (Plastic Eppendorf tubes are used throughout the process). The methanol was then washed for 20min twice in 100% DCM. Finally, samples were incubated (without shaking) in DiBenzyl Ether (DBE, Sigma 108014-1KG) until clear (about 30min) and then stored in DBE at room temperature. Organic solvents should be handled under a chemical hood, and disposed according to local health and safety regulations.

Light sheet imaging

Cleared samples were imaged in sagittal orientation (right lateral side up) on a light-sheet microscope (Ultramicroscope II, LaVision Biotec) equipped with a sCMOS camera (Andor Neo) and a 2X/0.5 objective lens (MVPLAPO 2x) equipped with a 6mm working distance dipping cap. Version v144 of the Imspector Microscope controller software was used. The microscope is equipped with LED lasers (488nm, 561nm and 640nm) with 3 fixed light sheet generating lenses. Scans were made at the 0.8X zoom magnification (1.6X effective magnification), with a light sheet numerical aperture of 0.1. Emission filters used are 525/50, 595/40 and 680/30. The samples were scanned with a step-size of 3µm using the continuous light sheet scanning method with the included contrast blending algorithm for the 640nm and 595nm channels (20 acquisitions per plane with a 50ms exposure), and without horizontal scanning for the 480nm channel (50ms exposure). To speed up the acquisitions, both channels where acquired in two separate scans.

Cresyl violet

For this second dataset, the full details are available in Vandenberghe et al., 2016 and in the PhD manuscript of a former student (Vandenberghe 2016 Sci Rep, https://theses.hal.science/tel-01381094).

Tissue processing

The fresh brain was snap frozen in isopentane (2-Methylbutane, Sigma-Aldrich) at -80°C. It was embedded in a mixture of M1 embedding matrix (Thermo Fisher Scientific) and Fast Green (Sigma-Aldrich), before being cut into 20-µm-thick serial coronal brain sections, from the frontal pole to the caudal end of the cerebral cortex, in a CM3050S cryostat (Leica). Sections were mounted on superfrost slides, quickly dried and stored at -80°C.

Histology

After careful thawing in +4°C PBS, one series was used for Nissl-staining (cresyl violet, Sigma-Aldrich) using a Shandon Varistain 24-4 automate (Thermo Electron Corporation) to achieve a reproducible stain.

Image acquisition

A flatbed scanner (ImageScanner III, G.E. Healthcare) was used to digitize the Nissl-stained sections (lateral resolution: 21 µm).

S1. Identification of the right *z-position* by an expert

In order to robustly and efficiently determine the *z-position* of all slices from a considered mouse brain, we asked an expert to identify, for each experimental slice I_r from a subset of slices, which slice $T_a \in B$ of the template was anatomically the closest. In other words, an expert was asked to visually match the experimental slices under consideration with those that were most similar in the template.

Let D be the set of experimental sections I_r describing one given mouse brain M_i. We took 15% of the total number of slices for each brain as a subset ($I_r \in D_{15\%}$), distributed equidistantly throughout the entire brain. This proportion was chosen as a compromise between a large enough number of slices considered to preserve robustness in estimating a ruling equation for all slices using a linear regression and as a limit of slices to consider due to the time-consuming, tedious and error-prone *z-position* manual identification task. A linear regression (LR) was thus applied on those estimated points. This resulted in the equation of the affine line connecting all slice numbers (a) of a given brain with that of the atlas template, *i.e.* the expert rating of the *z-position* of each of the slices (ER_z):

$$ER_z(I_r, B) = \underset{D_{15\%}}{\mathrm{LR}} \left[z(D_{15\%}, B) \right] = \gamma \, \frac{e_r}{e_t}(r-1) + \hat{t_1} \qquad \text{(s1)}$$

The equation results for the linear regressions $ER_z = f(r)$, as well as coefficients of determination (R^2) for each brain considered are presented in **Table s1** for autofluorescence (M_1-M_6) and cresyl violet (M_7-M_{13}) data respectively.

All R^2 values were significantly high: $R^2 > 0.99$ regardless of the brain or modality considered, which validated the use of the linear model and made the determination of the *z-position* of all the 2,771 slices, in the dataset presented in this article, robust and accurate.

S2. Realistic region-based histological protocols

Standardized conventional histological protocols for anatomical regions of different sizes were set up for each of the mouse brains studied. We considered six anatomical regions: cortex, striatum, thalamus, hippocampus, substantia nigra and globus pallidus. An expert was asked to identify, for each of these regions, the position of the first slice r_{start} where this anatomical region appeared, as well as the position of the last slice r_{end} including this region, into the experimental volume, along the antero-posterior axis. Based on these slice positions, a realistic sampling was proposed in order to generate analysis protocols in accordance with those performed in conventional routine. In particular, a typical inter-slice distance of 160 to 200 µm was chosen, according to the modality slice thickness. The example of landmarks identification for each of the six regions for the M_7 cresyl violet brain is presented in **Table s2**. Once the first slice number r_{start} and inter-slice distance d_{is} were defined, this gave a number of slices n to be considered per region, and it was then possible to extract the corresponding slices and treat them as a single and independent protocol. While keeping the number of slices considered and thus the inter-slice distance d_{is} fixed, the first slice considered was iterated to test another typical protocol for the same region. And so on until the last slice number r_{end} of the concerned region was reached. This enabled to test literally all the slices of a region in a given realistic multi-slices histological framework. Basically, d_{is}/e_r different protocols were tested for each region.

These were examples of conventional protocols targeting specific anatomical regions, they are clearly not exhaustive. They only served to make the proof of concept that the multi-slices extension allowed an accurate identification of *z-positions* in the classical study cases we can find in literature.

S3. Tilting angles estimation

To assess the two tilting angles that may occur when slicing mouse brains on a microtome compared to the atlas template (φ around the inferior-superior axis and β around the left-right axis), 3D reconstructed volumes from experimental brains were considered. The autofluorescence data considered are natively 3D (Renier at al., 2016). The cresyl violet sections were linearly registered to each other iteratively to reconstruct the 3D volume of the mouse brain using the Block Matching method (Ourselin et al., 2001; Vandenberghe et al., 2016).

Each of the 3D mouse brains considered was rigidly mapped to the 3D atlas template volume. From the resulting transformation matrix, it was possible to extract the two tilting angles φ and β.

The estimated values of these two angles φ and β are presented in **Table s3** for the autofluorescence (M_1-M_6) and cresyl violet (M_7-M_{13}) data respectively. They were all below 5°.

S4. Non-weighted dice score calculation

Let $ER(I_r)$ be the manual expert labeling of an experimental section I_r under consideration, thus containing the segmentation of each considered label c.

For a given label c and n experimental slices including this concerned label, the calculation of the mean dice score (DSc) between the atlas segmentation $\widehat{L}_{\hat{a}}$ and the expert labeling $ER(I_r)$ was defined as:

$$\overline{\mathrm{DSc}}^{(c)} = \frac{1}{n}\sum_{r=1}^{n} w^{(c)}\mathrm{DSc}(ER(I_r), \widehat{L}_{\hat{a}})^{(c)} \tag{s2}$$

with $w^{(c)}$ the weighting for each class, according to its support (number of pixels).

In this paper, the dice score was calculated in a non-weighted way, $w^{(c)}$ was thus equal for each class, independently of their support. We made this choice so that the final dice score for a given image reflected the ability of the method to segment brain slices, with respect to an expert, into regions regardless of their size (small and large regions).

S5. Estimation of the relative scaling factor (RSF) γ between the experimental data and the template

After estimating the *z-position* of each slice I_r from an experimental volume M_i considered, it was possible to estimate the RSF $\gamma_{\mathrm{M}i}$ between this data and the template:

$$\gamma_{\,\mathrm{M}_i} = \alpha(\mathrm{M}_i)\,\frac{e_t}{e_r(\mathrm{M}_i)} = \alpha\left[z(\mathrm{M}_i, B)\right]\,\frac{e_t}{e_r(\mathrm{M}_i)} \tag{s3}$$

with $\alpha(\mathrm{M}_i)$ the leading coefficient from the linear regression applied on the *z-positions* estimated between the slices I_r from M_i (thickness e_r) and the template slices T_a from B (thickness e_t).

Thus, the ratio $\eta_{\mathrm{M}i}$ between the theoretical RSF $\gamma_{theoric}$ and the one estimated by an expert γ_{expert} (expert rating) was calculated for each brain M_i:

$$\eta_{\,\mathrm{M}_i} = \frac{\gamma_{expert}(\mathrm{M}_i)}{\gamma_{theoric}(\mathrm{M}_i)} = \frac{\alpha_{expert}(\mathrm{M}_i)\,\frac{e_t}{e_r}}{\alpha_{theoric}(\mathrm{M}_i)\,\frac{e_t}{e_r}} = \frac{\alpha[ER_z(\mathrm{M}_i, B)]}{e_r(\mathrm{M}_i)} \tag{s4}$$

with e_r the thickness of the experimental sections I_r and M_i the different brains, i going from 1 to 13 here.

For a given modality *mod* (autofluorescence or cresyl violet here) including *N* brains under consideration, the calculation of γ_m was defined as the average of the different γ_{Mi} evaluated for each brain M_i:

$$\gamma_m(mod) = \frac{1}{N}\sum_{i}^{N}\gamma_{M_i}(mod) \qquad (s5)$$

Similarly, the calculation of η_m was defined as the average of the different η_{Mi} values for a given modality, such as:

$$\eta_m(mod) = \frac{1}{N}\sum_{i}^{N}\eta_{M_i}(mod) \qquad (s6)$$

The estimated RSF γ as well as the ratio η are presented in **Table s4** for autofluorescence (M_1-M_6) and cresyl violet (M_7-M_{13}) data, respectively.

For the autofluorescence, more specifically regarding the clearing protocol (iDISCO+), the average RSF between the six mouse half-brains and the template was estimated to be $\eta_m = 0.909 \pm 0.029$ (**Table s6**). The iDISCO+ protocol therefore led to a tissue shrinkage of about 9% along the antero-posterior axis compared to the template volume. This was coherent with literature as long as the template data was regarded as a reference with no change in volumetry compared to the native brain data. In other words, we considered that no modification of the sample size was introduced by *post mortem* treatments of the template sections digitized in this imaging modality to be able to compare our results to literature. Indeed, in the 3DISCO protocol (Ertürk et al., 2012), for which the successive improvements iDISCO (Renier et al., 2014) and iDISCO+ (Renier et al., 2016) were published, sample shrinkage (calculated on three brains) was estimated to be around 30% in 3D (Wan et al., 2018). As this shrinkage was considered spatially linear, it was possible to estimate the RSF on a single axis, such as $\eta = \sqrt[3]{1-0.3} = 0.888$; close to the value estimated for the six half-brains from M_1 to M_6. This meant that if this shrinkage was not taken into account ($\eta = 1$ by default), successive estimates of *z-positions* would include a deviation of about 9% propagated over each slice after identification of the first one (at each consideration of the distance d_t).

For the cresyl violet, more specifically regarding the whole histological protocol used in routine to obtain the M_7 to M_{13} data, the average RSF between the seven mouse brains and the template was estimated to be $\eta_m = 0.970 \pm 0.019$ (**Table s4**). This protocol therefore resulted in a relatively low tissue shrinkage of around 3% on average along the antero-posterior axis compared to the template volume.

In the multi-slices approach, we presented the respective results taking into account the different RSF $\gamma_{Mi} = \gamma_{expert}(M_i)$ per brain (*i* ranging from 1 to 13 here, **Equation s3**) on the one side, and the average RSF γ_m for one modality considered (**Equation s5**) on the other side.

S6. Easy-to-use interface

The giRAff method was implemented within the BrainVISA software platform. The different pipelines dedicated to the giRAff method and its extension giRAff_m for one and a series of histological slice(s) respectively benefited from the visualization tools and graphical user interfaces offered by the dedicated software. Examples of this interface for such pipelines are presented in **Figure S1**. No specific knowledge in neuroanatomy was mandatory for the use of this tool. Similarly, even if some technical parameters related to registration or high-performance computing appeared, these were all optional. Indeed, the general pipeline worked very well in an automated mode without any particular parameter settings. Only the pixel size and the experimental single slice thickness were required as

input parameters. Other optional parameters can be added, such as the number of computing cores to be used on the machine (**Figure S1A**) to benefit from high-performance computing solutions and save time, depending on its capacity (deployed on the SomaWorkflow tool). In the case of a multi-slices study, the inter-slice distance was required as an additional parameter (**Figure S1B**). Once more, the pipeline operated in an automated mode, but some users could benefit from their knowledge in neuroanatomy to tune parameters and refine the result. Indeed, by selecting one or several particular anatomical region(s) under study relative to the slices loaded in the pipeline as an input, the expert can save analysis time as it will pre-select the template slices corresponding to this or these region(s). Here, six regions of interest were specifically proposed: cortex, striatum, thalamus, hippocampus, globus pallidus and substantia nigra (**Figure S1B**).

REFERENCES

Ertürk, A., Becker, K., Jährling, N., Mauch, C. P., Hojer, C. D., Egen, J. G., et al. (2012). Three-dimensional imaging of solvent-cleared organs using 3DISCO. *Nat. Protoc.* 7, 1983–1995. doi: 10.1038/nprot.2012.119

Ourselin, S., Roche, A., Subsol, G., Pennec, X., and Ayache, N. (2001). Reconstructing a 3D structure from serial histological sections. *Image Vis. Comput.* 19, 25–31. doi: 10.1016/S0262-8856(00) 00052-4

Renier, N., Wu, Z., Simon, D. J., Yang, J., Ariel, P., and Tessier-Lavigne, M. (2014). iDISCO: a simple, rapid method to immunolabel large tissue samples for volume imaging. *Cell* 159, 896–910. doi: 10.1016/j.cell.2014.10.010

Renier, N., Adams, E. L., Kirst, C., Wu, Z., Azevedo, R., Kohl, J., et al. (2016). Mapping of brain activity by automated volume analysis of immediate early genes. *Cell* 165, 1789–1802. doi: 10.1016/j.cell.2016.05.007

Vandenberghe,M. E.,Hérard, A. S., Souedet,N., Sadouni, E., Santin,M.D., Briet,D., et al. (2016). High-throughput 3D whole-brain quantitative histopathology in rodents. *Sci. Rep.* 6, 20958. doi: 10.1038/srep20958

Wan, P., Zhu, J., Xu, J., Li, Y., Yu, T., and Zhu, D. (2018). Evaluation of seven optical clearing methods in mouse brain. *Neurophotonics* 5, 035007. doi: 10.1117/1.NPh.5.3.035007

TABLES AND FIGURES

TABLE s1 | Equation lines ER_z, as well as their corresponding coefficient of determination R^2 estimated by linear regression after expert pairing between each experimental volume considered (15% of all slices equidistantly spaced) and the template slices for the six autofluorescence mouse brains (M_1-M_6) and the seven cresyl violet mouse brains (M_7-M_{13}).

**AUTOFL**	M_1	M_2	M_3	M_4	M_5	M_6	
ER_z	$0.223r + 7.9$	$0.215r + 12.2$	$0.234r + 11.6$	$0.230r + 10.9$	$0.233r + 11$	$0.229r + 8.07$	
R^2	0.998	0.999	0.999	0.998	0.999	0.997	
**CRESYL**	M_7	M_8	M_9	M_{10}	M_{11}	M_{12}	M_{13}
ER_z	$0.776r + 21.0$	$0.781r + 12.1$	$0.777r + 19.3$	$0.766r + 20.1$	$0.787r + 18.2$	$0.748r + 19.8$	$0.796r + 15.8$
R^2	0.996	0.999	0.995	0.998	0.998	0.997	0.996

TABLE s2 | Start-end slice numbers, length and number of considered slices considering an inter-slice distance of 160 µm in six main anatomical regions (cortex, striatum, thalamus, hippocampus, globus pallidus and substantia nigra) in realistic multi-slices histological protocols for the cresyl violet mouse brain M_7.

**Cresyl violet M_7**	**cortex**	**striatum**	**thalamus**	**hippo-campus**	**globus pallidus**	**substantia nigra**
r_{start} - r_{end} (slice number)	6 - 93	18 - 70	42 - 87	49 - 91	39 - 54	65 - 84
**Length** (nb of slices)	87	52	45	42	15	19
**Length** (µm)	6 960	4 160	3 600	3 360	1 200	1 520
n (nb of slices)	**44**	**24**	**18**	**20**	**7**	**6**

TABLE s3 | Tilting angles φ around the infero-superior axis and β around the left-right axis estimated on the six autofluorescence mouse brains (M_1-M_6) and the seven cresyl violet mouse brains (M_7-M_{13}) and the seven cresyl violet mouse brains (M_7-M_{13}), as well as the average and standard deviation values.

**AUTOFLUO**	**M_1**	**M_2**	**M_3**	**M_4**	**M_5**	**M_6**	**MEAN**	
**φ (°)**	*0.01*	0.00	0.00	0.00	0.00	0.00	**0.00 ± 0.01**	
**β (°)**	0.00	0.00	0.00	*0.08*	0.00	0.00	**0.01 ± 0.03**	
**CRESYL**	**M_7**	**M_8**	**M_9**	**M_{10}**	**M_{11}**	**M_{12}**	**M_{13}**	**MEAN**
**φ (°)**	*0.04*	*0.18*	0.00	*3.31*	*0.06*	*0.17*	*0.24*	**0.57 ± 1.21**
**β (°)**	*0.03*	0.00	0.00	*0.04*	*1.78*	*0.32*	*0.08*	**0.32 ± 0.65**

TABLE s4 | RSF between the autofluorescence brains (M_1-M_6) - the seven cresyl violet mouse brains (M_7-M_{13}) and the template, as well as the ratio between the RSF estimated by the expert thanks to the ER_z (γ_{expert}) and the theoretical RSF given by the slice thickness e_r ($\gamma_{theoric}$).

**AUTOFL**	**M_1**	**M_2**	**M_3**	**M_4**	**M_5**	**M_6**	**MEAN**	
γ_{expert}	0.223	0.215	0.234	0.230	0.233	0.229	0.227 ± 0.007	
$\gamma_{theoretical}$	0.250	0.250	0.250	0.250	0.250	0.250	0.250	
**η**	**0,892**	**0,860**	**0,936**	**0,920**	**0,932**	**0,916**	**0,909 ± 0.029**	
**CRESYL**	**M_7**	**M_8**	**M_9**	**M_{10}**	**M_{11}**	**M_{12}**	**M_{13}**	**MEAN**
γ_{expert}	0,776	0,781	0,777	0,766	0,787	0,748	0,796	0.776 ± 0.015
$\gamma_{theoretical}$	0.800	0.800	0.800	0.800	0.800	0.800	0.800	0.800
**η**	**0,970**	**0,976**	**0,971**	**0,958**	**0,984**	**0,935**	**0,995**	**0,970 ± 0.019**

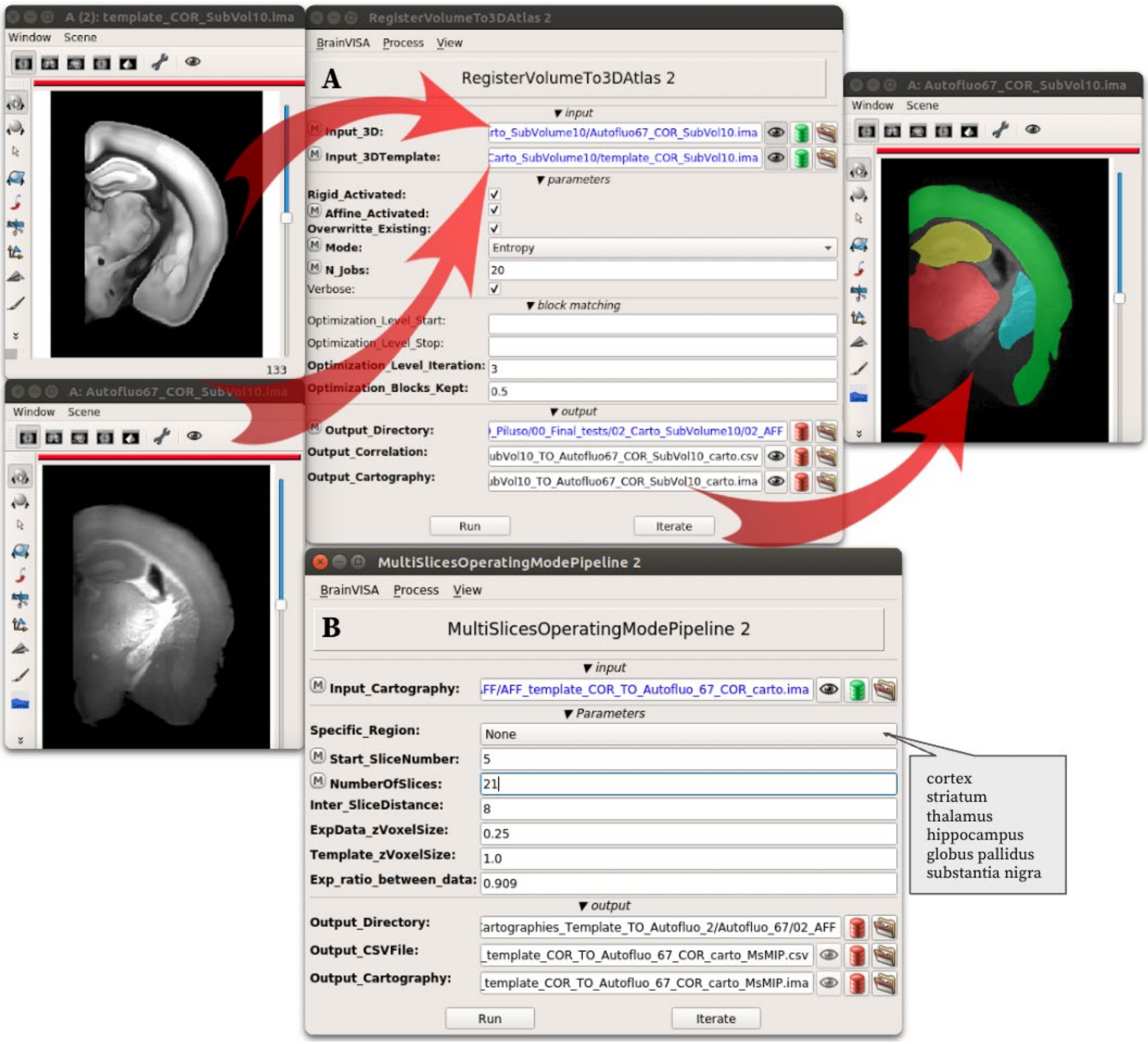

FIGURE S1 | Examples of giRAff sub-pipelines implemented in the BrainVISA software platform (https://brainvisa.info) for a given experimental mouse half-brain single slice (A) for the giRAff method and (B) for the giRAff$_m$ extension.
